# Toward a Novel Multilocus Phylogenetic Taxonomy for the Dermatophytes

**DOI:** 10.1007/s11046-016-0073-9

**Published:** 2016-10-25

**Authors:** G. Sybren de Hoog, Karolina Dukik, Michel Monod, Ann Packeu, Dirk Stubbe, Marijke Hendrickx, Christiane Kupsch, J. Benjamin Stielow, Joanna Freeke, Markus Göker, Ali Rezaei-Matehkolaei, Hossein Mirhendi, Yvonne Gräser

**Affiliations:** 1 CBS-KNAW Fungal Biodiversity Centre, Utrecht, The Netherlands; 2 Institute of Biodiversity and Ecosystem Dynamics, University of Amsterdam, Amsterdam, The Netherlands; 3 Basic Pathology Department, Federal University of Paraná State, Curitiba, Paraná Brazil; 4 Peking University Health Science Center, Research Center for Medical Mycology, Beijing, China; 5 Shanghai Institute of Medical Mycology, Changzheng Hospital, Second Military Medical University, Shanghai, China; 6 Biological Sciences Department, Faculty of Science, King Abdulaziz University, Jeddah, Saudi Arabia; 7 Department of Dermatology, Centre Hospitalier Universitaire Vaudois, Lausanne, Switzerland; 8 Mycology and Aerobiology, Scientific Institute of Public Health, Brussels, Belgium; 9 Institute of Microbiology and Hygiene, University Medicine Berlin - Charité, Berlin, Germany; 10 Thermo Fisher Scientific, Landsmeer, The Netherlands; 11 Leibniz Institute DSMZ-German Collection of Microorganisms and Cell Cultures, Brunswick, Germany; 12 Health Research Institute, Infectious and Tropical Diseases Research Center, Ahvaz Jundishapur University of Medical Sciences, Ahvaz, Iran; 13 Department of Medical Mycology, School of Medicine, Ahvaz Jundishapur University of Medical Sciences, Ahvaz, Iran; 14 Department of Medical Parasitology and Mycology, School of Public Health, Tehran University of Medical Sciences, Tehran, Iran

**Keywords:** *Arthrodermataceae*, Dermatophytes, Phylogeny, Taxonomy, *Trichophyton*

## Abstract

Type and reference strains of members of the onygenalean family *Arthrodermataceae* have been sequenced for rDNA ITS and partial LSU, the ribosomal 60S protein, and fragments of β-tubulin and translation elongation factor 3. The resulting phylogenetic trees showed a large degree of correspondence, and topologies matched those of earlier published phylogenies demonstrating that the phylogenetic representation of dermatophytes and dermatophyte-like fungi has reached an acceptable level of stability. All trees showed *Trichophyton* to be polyphyletic. In the present paper, *Trichophyton* is restricted to mainly the derived clade, resulting in classification of nearly all anthropophilic dermatophytes in *Trichophyton* and *Epidermophyton*, along with some zoophilic species that regularly infect humans*. Microsporum* is restricted to some species around *M. canis*, while the geophilic species and zoophilic species that are more remote from the human sphere are divided over *Arthroderma, Lophophyton* and *Nannizzia*. A new genus *Guarromyces* is proposed for *Keratinomyces ceretanicus*. Thirteen new combinations are proposed; in an overview of all described species it is noted that the largest number of novelties was introduced during the decades 1920–1940, when morphological characters were used in addition to clinical features. Species are neo- or epi-typified where necessary, which was the case in *Arthroderma curreyi*, *Epidermophyton floccosum*, *Lophophyton gallinae*, *Trichophyton equinum*, *T. mentagrophytes*, *T. quinckeanum*, *T. schoenleinii*, *T. soudanense*, and *T. verrucosum*. In the newly proposed taxonomy, *Trichophyton* contains 16 species, *Epidermophyton* one species, *Nannizzia* 9 species, *Microsporum* 3 species, *Lophophyton* 1 species, *Arthroderma* 21 species and *Ctenomyces* 1 species, but more detailed studies remain needed to establish species borderlines. Each species now has a single valid name. Two new genera are introduced: *Guarromyces* and *Paraphyton*. The number of genera has increased, but species that are relevant to routine diagnostics now belong to smaller groups, which enhances their identification.

**Electronic supplementary material:**

The online version of this article (doi:10.1007/s11046-016-0073-9) contains supplementary material, which is available to authorized users.

## Introduction

The dermatophytes belong to the oldest groups of microorganisms that have been recognized as agents of human disease. The taxonomy of these fungi was initiated in 1841 with the studies of Robert Remak and David Gruby [1]. Between 1840 and 1875, five of the main species known today, viz. *Microsporum audouinii*, *Epidermophyton floccosum*, *Trichophyton schoenleinii*, *T. tonsurans* and *T. mentagrophytes* had already been described; this was several decades before the discovery of Pasteur’s invention of axenic culture [2]. The only ubiquitous modern dermatophyte missing from the list is *Trichophyton rubrum* [3], which has been hypothesized to have emerged in the twentieth century [4].

**Fig. 1:**
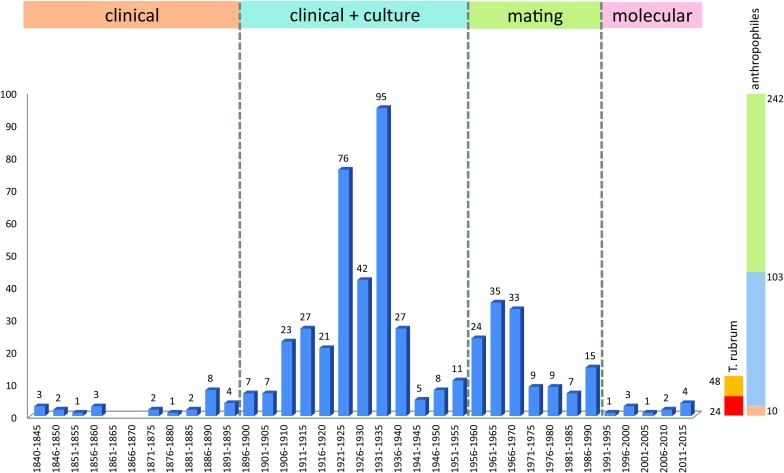
Number of name changes of members of *Arthrodermataceae* during the period 1840–2015, with 5-year increments. The largest number of new names was created when morphology was added to clinical data as criteria for species distinction. The period 1960–1995 is marked by the addition of teleomorph names, leading to dual nomenclature of the dermatophytes. The *bar* at the *right* shows the approximate number of existing anthropophilic species (*n* = 10), the number of times these have been described (basionyms: *n* = 103) and the total number of name changes for these 10 species (*n* = 242). Possible [7] and proven synonyms of *Trichophyton rubrum* are listed in ocher (*n* = 48), of which (*n* = 24) were basionyms, in *red*

After Pasteur’s time, culturing of dermatophytes and description of new species has taken off enormously. Species were defined on the basis of combined clinical pictures and morphological characters in vitro. Sixteen human-associated species were introduced between 1870 and 1920, with Sabouraud’s [5] magistral overview of the dermatophytes setting a new standard. During the decades that followed, application of the new methodological standard led to an explosion of new species and recombined names (Fig. 1). Generic concepts remained confused, leading to repeated name changes with a total of 350 names around the year 1950. Subsequently anamorph nomenclature stabilized by the wide acceptance of *Epidermophyton*, *Microsporum* and *Trichophyton* as the genera covering all dermatophytes.

Culture and microscopic morphology worked well as diagnostic parameters when fresh isolates were used, but were difficult to maintain and reproduce because of rapid degeneration. Standardization with reference strains was therefore difficult, and this led to the introduction of numerous taxa that are now regarded as synonyms of earlier described species. In addition, diverse types of morphological mutants were described as separate taxa, such as *Keratinomyces longifusus*, which turned out to be *Microsporum fulvum* with strongly coherent conidia [6]. This misclassification is an unavoidable consequence of a diagnostic system based on the phenotype. Similar misjudgments of mutants of a single species also occurred elsewhere, sometimes unknowingly leading to the description of a separate genus for the mutant: compare, e.g., the genus pairs *Bipolaris*/*Dissitimurus*, *Scedosporium*/*Polycytella*, *Exophiala*/*Sarcinomyces*, or *Trichosporon*/*Fissuricella* [7]. In addition, several dermatophytes are known which do not or poorly sporulate in culture and thus show very limited phenotypic characteristics. Classically such species were partly based on clinical symptoms, e.g., *T. concentricum* or *T. schoenleinii*, but many more, undescribed species may exist [8].

In the last decades of the twentieth century, it became obvious that morphology had its limitations and could not be used as sole characteristic for classification or identification. Given these problems, Weitzman et al. [9] introduced an additional character set in the form of physiological parameters, so-called trichophyton-agars utilizing the ability of strains to assimilate a panel of essential vitamins, but also growth temperature, gelatin liquefaction, etc. The method now indicated as the ‘conventional approach’ to dermatophyte taxonomy combines clinical appearance, cultural characteristics, microscopy and physiology. Serology has never really taken off.

Biological species concepts entered the picture with the modern rediscovery of dermatophyte teleomorphs by Dawson and Gentles [10] and Stockdale [11]. Several geophilic and zoophilic dermatophytes, as well as related non-pathogenic species like *Trichophyton terrestre* and *T. ajelloi*, were found to produce sexual states, for which the genera *Arthroderma* and *Nannizzia* were introduced. This led to a new boom in the number of names (Fig. 1) and marked the introduction of dual nomenclature for dermatophytes. The delineation of sexual interaction began to take an unusual course when Stockdale [12] discovered that members of many apparently non-mating species could be induced to reveal their mating type in an incomplete mating reaction with testers of *Arthroderma simii*. Most of the recognized asexual species could be typed in this manner and demonstrated to be descended from a single ancestral mating type. For example, *Trichophyton rubrum* was shown to be (−) in mating type, while its close relative *T. megninii*, currently considered to be synonymous, was (+). Just a few important species, such as *Epidermophyton floccosum* and *T. soudanense*, a further member of the rubrum series, resisted typing with this system and remained of unknown status. Summerbell [13] pointed out the obvious ecological factor linking the non-sexual species: they all infected animals (including *Homo sapiens*) without having a terrestrial reservoir allowing the elaborate sexual processes with ascigerous fruit bodies to take place on keratinous debris.

### Clinical Significance

Large differences are known to exist between species with respect to their natural habitat. Three broad ecological groups of dermatophyte species are recognized: anthropophilic, zoophilic, or geophilic (Table 1). Sometimes species cannot be clearly attributed to one of these groups due to insufficient data. Anthropophilic species naturally colonize humans, being transmitted between humans and usually cause chronic, mild, non-inflammatory infections and often reaching epidemic proportions. Animal-carriage of these species does occur [14] but is exceptional. Zoophilic species live in close association with animals other than humans and transmission to humans usually occurs through their reservoirs. The fungi occur in the fur of particular animal hosts, either symptomatically or asymptomatically, and can become epidemic. Geophilic dermatophytes have their reservoir in the soil around burrows of specific terrestrial mammals, feeding on keratinous debris. They may be carried by these animals in their fur [15]; hence, the difference between geophilic and zoophilic dermatophytes is not always sharp. When transmitted to humans, zoo- and geophilic species cause acute, inflammatory mycoses. Occasionally, humans infected by zoophiles remain contagious, leading to small, self-limiting outbreaks [16], while most infections by geophiles are quickly resolved. Thus, also in the effectivity of human-to-human transmission an increasing trend is observed from geophiles via zoophiles to anthropophiles. No sexual phases are known in truly anthropophilic species, while geophilic species show vigorous mating. By these combined parameters, the three ecological groups, although not sharply separated, are fundamentally different and also have clinical significance (Table 1).

**Table 1:** Broad classification of dermatophytes on the basis of ecological and clinical parameters

	Geophilic	Zoophilic	Anthropophilic
Phylogeny	Ancestral	Moderate	Derived
Sexuality	Vigorous mating	Mostly mating	Clonal
Infection	Highly inflammatory	Moderately inflammatory	Non-inflammatory
Transmission	Via environment	Double life cycle	Via host
Resolution	Quickly resolved	Resolved, self-limiting epidemics	Chronic

### Experimental Methods

Enabled by the recent publication of whole genome sequences of several dermatophyte species [17], idiomorphs of the mating type loci (alpha domain and HMG domain genes) were detected directly at DNA level. Using partial amplification of each locus, Kano et al. [18] were able to confirm molecularly that 22 *T. verrucosum* strains exhibited a single mating type only. Gräser et al. (unpublished data) revealed that a single mating type was present in numerous species: *T. tonsurans*, *T. equinum*, *T. interdigitale*, *T. schoenleinii*, *T. rubrum*, *T. violaceum*, *T. erinacei*, *T. concentricum*, *M. audouinii* and *M. ferrugineum*. This supports the view of clonal reproduction due to the loss of one of the mating types on species level. Some exceptions were found with zoophilic species such as *T. benhamiae* and *T. mentagrophytes*, where both types such as alpha and HMG were present with different distribution ratios [19, 20]. This implies that all anthropophilic and most zoophilic dermatophytes reproduce clonally by asexual propagation in apparently stable environmental niches. In contrast, Anzawa et al. [21] showed mating of a highly competent *A. simii* tester strain producing a fertile F1 generation with a strain of *T. rubrum*, challenging the biological species concept, although only a single out of 35 ascospores proved to be a real hybrid of the two species. Apparently, the dermatophytes have held an atavistic ability to undergo genetic exchange via sexual reproduction/hybridization in response, e.g., the stressful conditions of a newly inhabited environment. In practice, due to the different ecological niches of species like the anthropophilic species *T. rubrum* and the zoophilic species *A. simii,* they do not have the possibility to meet each other in nature.

Like in Pasteur’s days, when axenic culture revolutionized microbiology, the application of molecular methods since 20 years has revolutionized dermatophyte taxonomy and that of other fungi. First molecular papers used the ribosomal small and large subunits as markers [22, 23]. In a series of papers, Gräser et al. [6, 24] applied the more variable rDNA ITS region and were able to resolve a large number of species. This molecular system has been confirmed several times in later studies [25] and with different molecular markers such as *BT2* [26, 27] and *TEF1* [28]. The main topology of the *Arthrodermataceae* seems to be molecularly stable, but does not entirely correspond with morphology, as *Trichophyton* appears to be polyphyletic. As noted in earlier papers by Gräser et al. [6, 24], anthropophilic species are confined to some derived clusters, zoophilic species of domesticated mammal hosts are located in the middle of the tree, while geophilic species are located in an ancestral position, and the lower clusters are still unstable due to taxon sampling effects. For reasons of clinical understanding, it is recommendable to formalize these differences in a new taxonomic system, which is one of the aims of the present paper.

While the molecular approach was able to resolve the main traits of dermatophyte evolution, it may fail in the details. Several well-established, clinically different species such as *Trichophyton rubrum*/*T. violaceum, T. equinum*/*T. tonsurans* and to a certain extent also *M. audouinii*/*M. canis*/*M. ferrugineum* appeared largely indistinguishable in our multilocus analysis. Small sequencing ambiguities or missing data in this large dataset may blur the small differences very recently emerged species. Therefore, despite the available large body of research on these species, polyphasic studies combining molecular, ecological, phenotypic and life cycle data are needed to establish the validity of these species with certainty.

**Fig. 2:**
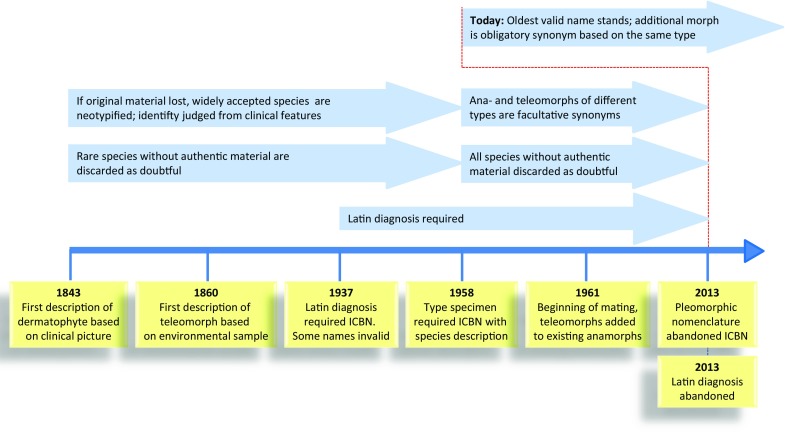
Overview of changing taxonomic principles during the period 1840–2015. Of the oldest species, no original material has been preserved; the rare ones are discarded as being doubtful; the widely used names are neotypified. Latin diagnoses were required between 1937 and 2013. Pleomorphic naming with separate typification of ana- and teleomorph has been relevant between 1957 and 2013. The generic and specific nomenclatural system proposed in the present article is valid from the situation per January 1, 2013 onwards

With the various taxonomic approaches, also nomenclatural rules have evolved over time (Fig. 2). In the nineteenth century, a clinical description was judged sufficient to characterize a fungus. Deposition of a type specimen became compulsory only in 1957. Today, the reference of a type is essential to stabilize the species’ delimitation and nomenclature. Older, long-forgotten names without types are discarded as doubtful, but well-known species names should be maintained by neotypification [6]. During the decades of dual nomenclature, species can have two types, but since 2013 the name, anamorph or teleomorph, always refers to the same, original type specimen. Present-day naming of fungi is according to their gross phylogenetic position. It should be realized, however, that positions in trees are relative, being dependent on the coincidentally selected constituents of the tree. Therefore, polyphasic species remain concepts essential for reliable nomenclature.

For a checklist of obsolete names in dermatophytes for which no type material is known to exist, is referred to de Hoog et al. [7]. Numerous later-described species were placed in synonymy, because they proved to not to deviate on the basis of modern characters. de Hoog et al. [7] listed 24 basionyms (with 48 combinations in total) as probably synonymous with *Trichophyton rubrum* (Fig. 1) (although only 5 basionyms could be proven with extant type materials). Several of the apparent synonyms were only recently segregated from *T. rubrum* on the basis of physiological parameters, which has shed doubt over usefulness of physiology as a taxonomic parameter.

## Materials and Methods

### Nomenclature

A search for possible generic names in *Arthrodermataceae* was limited to members of the order *Onygenales*. Candidate generic names were those type species in the family according to the Index Fungorum (www.indexfungorum.org). Obsolete generic names were taken from species synonyms and list of doubtful species in the *Atlas of Clinical Fungi* [7]. For every taxon to be accepted as a potential name or synonym, permanently inactivated (dried or under liquid nitrogen) holotype material had to be necessary. Holotypes as well as living strains connected with the holotypes were indicated as type (T). In heterothallic species, mating partners needed to obtain the teleomorph were listed as syntypes (ST). Taxa without types were discarded as doubtful, or, when these concerned well-known clinical taxa described without deposition of type material, were neotypified. Neotypes (NT) in the present article have a single CBS (Centraalbureau voor Schimmelcultures) number, which refers to dried holotype material, or to metabolically inactivated samples under liquid nitrogen of which the original batch will remain unopened. In case the original holotype may not be interpretable, epitypes (ET) were indicated. If no type was indicated in the original protologue, but strains from the describing author(s) were available, these were listed as authentic (AUT). If none of these applies, but strains were used by authoritative authors, they were listed as reference strains. The latter two categories do not have official nomenclatural status.

### Strains Analyzed

Strains preserved in the reference collection of Centraalbureau voor Schimmelcultures (CBS-KNAW Fungal Biodiversity Centre) were used for the multilocus phylogenetic analysis of members of the family *Arthrodermataceae*. In total, 261 strains were analyzed. Strains were cultured on Sabouraud’s glucose agar (SGA) plates using lyophilized, cryo-preserved or fresh mycelial material for inoculation. Most of the cultures were incubated for 7 to 14 days at the temperature of 24 °C, with some exceptions for very slow-growing species, while some others grew within a few days.

### DNA Extraction, PCR and Sequencing

Genomic DNA was isolated from either preserved material or material harvested from living cultures. The DNA extraction was performed using MasterPure™ Yeast DNA Purification Kit from Epicentre. Five gene regions were amplified: ITS and LSU loci of the rDNA operon [29] and two protein coding genes. The universal fungal locus ITS1-5.8-ITS2 of the rDNA was amplified with ITS5 [30] and ITS4 [31] according to standard protocols [32]. The D1-D2 region of LSU was amplified using primers LR0R and LR5 [33] according to conditions as for ITS except for a longer extension time (90 s). Partial β-tubulin (*TUB*) was amplified with primers TUB2Fd and TUB4Fd [34]. PCR had an annealing temperature of 58 °C for one min and elongation time of 70 s. 60S ribosomal protein L10 was amplified with 60S-908R and 60-S506F [35].

All PCRs were done in 12.5 μL final PCR volume (CBS-KNAW barcoding lab protocol), using 2.5 μL of the DNA extract, 1.25 μL PCR buffer (Takara, Japan, incl. 2.5 mM MgCl_2_), 1 μL dNTPs (1 mM stock; Takara, Japan), 0.6 μL dimethylsulfoxide (DMSO; Sigma, The Netherlands), forward-reverse primer 0.25 μL each (10 mM stock), 0.06 μL (5 U) Takara HS Taq polymerase, 7.19 μL MilliQ water [32, 36]. PCR products were visualized on 1 % agarose gel. Positive PCR products were sequenced in cycle-sequencing reaction using ABI big dye terminator v.3.1 using only one quarter of the suggested volume (modified manufacturer’s protocol). Bidirectional sequencing was performed in a capillary electrophoresis system (Life Technologies 3730XL DNA analyser). The obtained sequences were manually edited, and consensus sequences were stored in a Biolomics database [37].

### Sequence Alignment and Phylogenetic Analysis

**Fig. 3:**
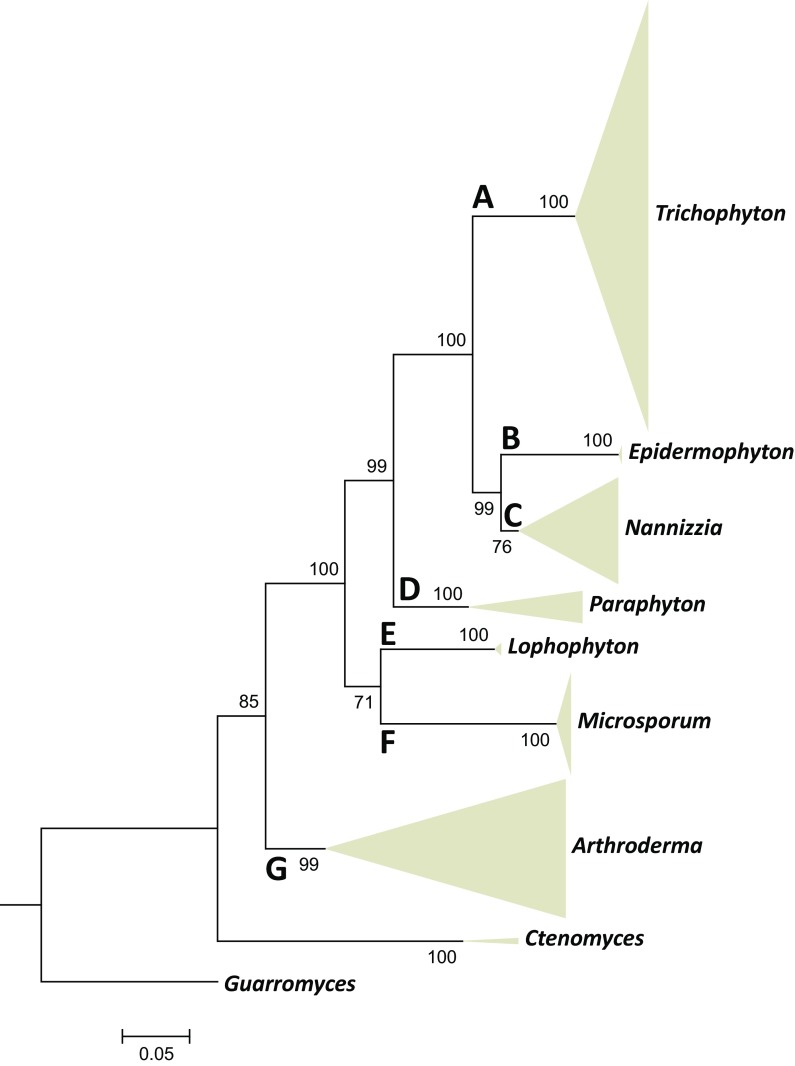
Maximum likelihood phylogenetic tree (RAxML v.8.0.0) based on ITS and partial LSU, *TUB* and 60S L10 sequences of *Arthrodermataceae* species using GTRCAT as model, with 1000 bootstrap replications, shown when >70 %, where genera were collapsed. *Guarromyces ceretanicus* was selected as outgroup

**Fig. 4:**
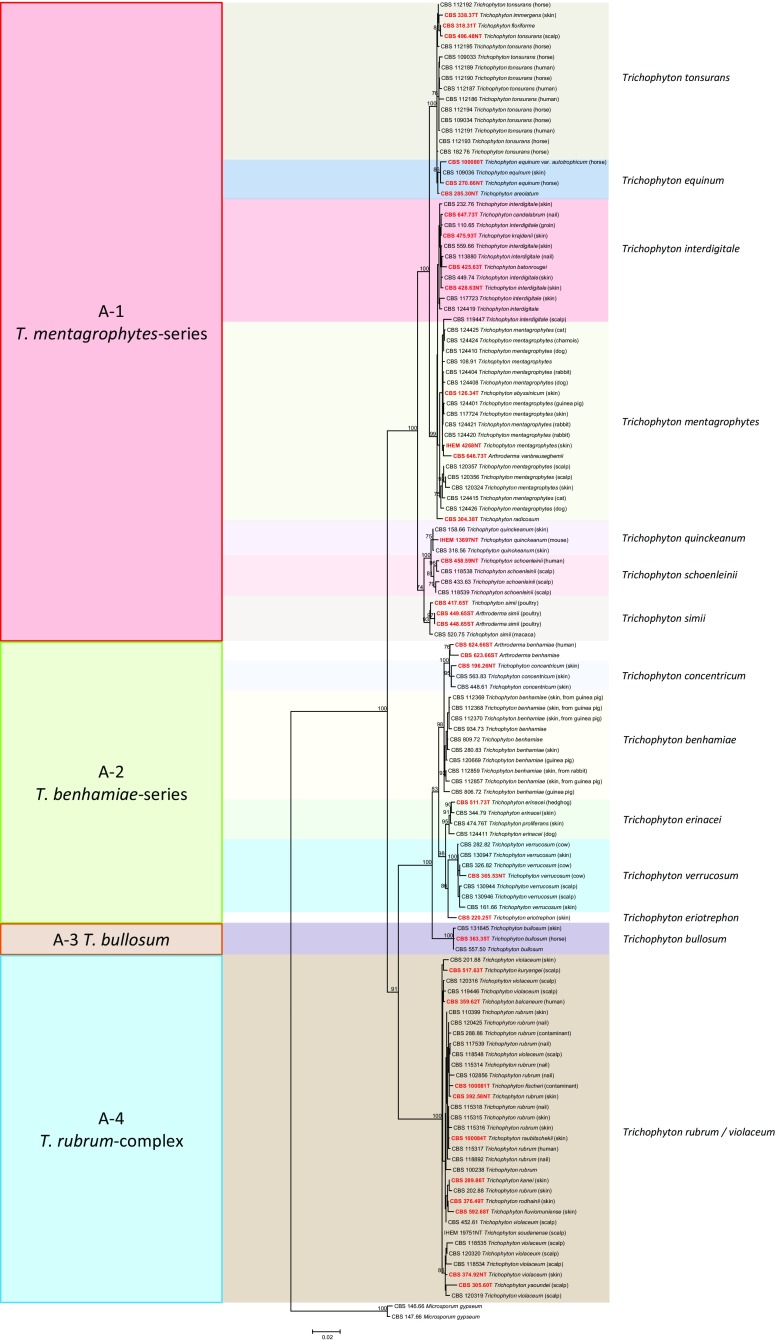
Maximum likelihood phylogenetic tree (RAxML v.8.0.0) based on ITS and partial LSU, *TUB* and 60S L10 sequences of *Trichophyton* species using GTR + GI as model, with 1000 bootstrap replications, shown when >70 %. *Microsporum gypseum* was selected as outgroup

Sequences were aligned with MAFFT v. 6.850b using default settings except for the ‘genafpair’ option [38]. The datasets for the five loci were assembled in one multilocus dataset using sequence matrix software and deposited in Genbank. Alignments were compared manually and via the Gblocks server (http://molevol.cmima.csic.es/castresana) with stringency settings ‘allow gaps positions within the final blocks’ and ‘do not allow many contiguous nonconserved positions’. For both ITS and multilocus dataset Maximum likelihood phylogeny was inferred using RAxML v. 8.0.0 employing GTRCAT model and 1000 bootstrap replicates. Bootstrap branch supports above 80 % are shown. A general rDNA ITS and several more detailed multilocus single-genus trees are provided in summary (Figs. 3, 4).

## Results and Discussion

**Fig. 5:**
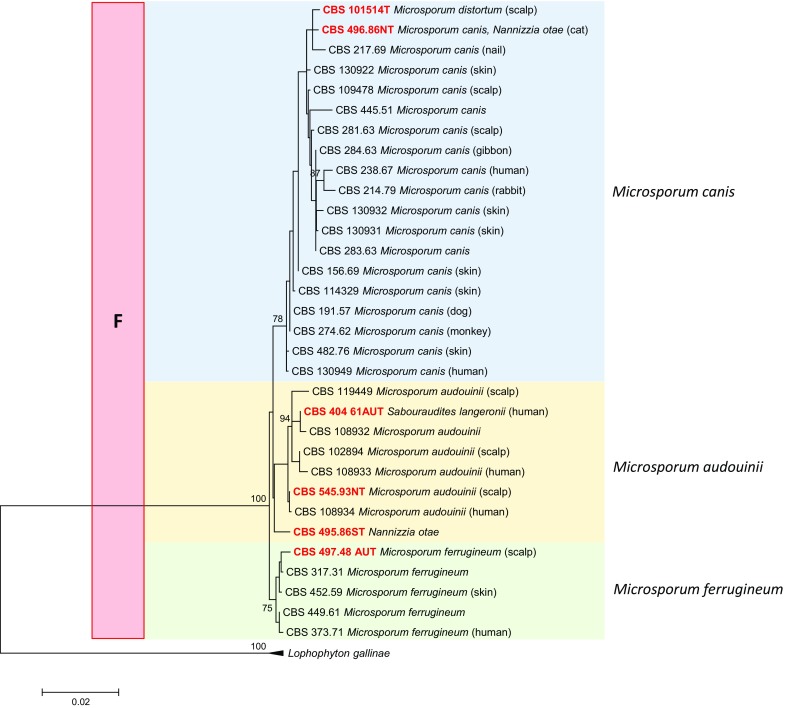
Maximum likelihood phylogenetic tree (RAxML v.8.0.0) based on ITS and partial LSU, *TUB* and 60S L10 sequences of *Microsporum* species using T92 + G as model, with 1000 bootstrap replications, shown when >70 %. *Arthroderma grubyi* was selected as outgroup

A phylogenetic tree was constructed for all species discussed in this paper using the ITS rDNA region only, since this gene was comparable and alignable over the entire set of strains (Fig. 3). Seven clades were distinguishable. The upper clade (A) in this figure comprised anthropophilic and zoophilic *Trichophyton* species. This clade is shown in more detail with multilocus data in Fig. 4. Four 100 % bootstrap-supported species or species series were recognizable: (A-1) *Trichophyton mentagrophytes* and related anthropophilic and zoophilic species including some strictly anthropophilic clonal offshoots, with *Trichophyton interdigitale* and *T. tonsurans* as most common species; (A-2) *Trichophyton benhamiae* series with *T. schoenleinii* and *T. verrucosum*; (A-3) The zoophilic species *Trichophyton bullosum*; (A-4) *Trichophyton rubrum* series in which no individual species could be distinguished. The next, well-supported clade (B) in Fig. 1 contained a single species, *Epidermophyton floccosum*, which is paraphyletic to clade (C). Clade (C) contained zoophilic and geophilic species of which *Microsporum gypseum* was the most common one. Clades (D) and (E) were two groups of large-conidial, heterothallic species. Clade (F) comprised the *Microsporum canis* series, which is shown in more detail with multilocus data in Fig. 5. Clade (G) was highly diverse, containing well-resolved geophilic species only, many of which are currently known under their *Arthroderma* teleomorph name because of heterothallic mating. The anamorphs were characterized by large, multi-celled, thick- and rough-walled macroconidia and abundant microconidia.

Data were also generated for additional partial genes LSU, 60S L10, and *TUB* (Figs. 4, 5). Clades (A) and (F), containing the great majority of species that are relevant in clinical and veterinary settings, were partially resolved. A number of classical species in medical and veterinary mycology proved to be indistinguishable, possibly due to the fact that the large number of SNPs overshadowed consistent differences. The application of the Gblocks tool, reducing ambiguously aligned positions, led to inclusion of only 39 % of the original 830 positions in ITS and reduced the resolution between species. For this reason, we maintained manually aligned datasets and used additional phenotypic and ecological data for species delimitation. This did not always yield expected results; further detailed studies with mating tests remain necessary. In this study we differentiate ‘species series’, which are larger clusters of taxa which unite at the ITS level, and ‘species complexes’. Chen et al. [39] defined a complex as a number of populations that are doubtfully distinct. In our species series, some of the taxa were unambiguously different when multilocus data were applied, while neighboring taxa could not properly be distinguished and thus might be regarded as species complexes. For precise species delimitation, data on natural hosts, virulence on non-optimal hosts, growth and sporulation, metabolite production and mating behavior are needed in addition to more detailed molecular studies. In the present overview, we prefer to be conservative in the maintenance of the number of species until more precise studies have proven exact borderlines of biological species and more understanding of host-specificity is acquired.

### The Species Problem

In the *T. mentagrophytes* series (Clade A-1) in Fig. 4 showing a multilocus tree, *T. mentagrophytes* was close to *T. interdigitale*. The latter species was exclusively isolated from humans, while *T. mentagrophytes* preponderantly originated from animals but also contained clinical strains. *Trichophyton equinum* could as yet not be distinguished from *T. tonsurans*. This touches on an essential question in medical mycology, as the species couples are known as zoophilic and anthropophilic, respectively, and a human infection by a zoophile is believed to be more inflammatory than when there is no host change. These questions cannot be solved in the present overview due to lack of clinical data of the strains examined. In the *T. benhamiae* series (Clade A-2), *Trichophyton benhamiae*, *T. concentricum*, *T. erinacei* and *T. verrucosum* could all be separated with multilocus data. *Trichophyton quinckeanum* is very close to *T. schoenleinii*. The *Trichophyton rubrum* complex (Clade A-4) showed some diversity, but this did not entirely match with observed differences in phenotype and clinical predilection. In clade (F), when analyzed with multilocus data (Fig. 5), *Microsporum canis*, *M. audouinii* and *M. ferrugineum* were difficult to distinguish, particularly because the (+) and (−) mating partners showed a mutual distance that spanned the diversity of nearly the entire genus. With distance, a gradational loss of sporulation is observed via an ‘*M. distortum* phenotype’, concomitant with adaptation to the human host, which is in accordance with current species concepts.

A major taxonomic problem, frequently encountered in environmental fungi in general, is unexpected phylogenetic diversity of groups that previously seemed to be phenotypically monomorphic. Species with similar microscopic appearance sometimes even prove to belong to entirely different orders. Dermatophytes, in contrast, have consistently been found to belong to a single lineage, i.e., the family *Arthrodermataceae*. This shared phylogeny has been explained by their keratinophilic character, which is a rare property in the fungal kingdom. Evolution within the family shows a strong coherence with the animal hosts providing the keratin, as already noted in classical literature [40].

A second, current taxonomic problem is the molecular species concept. Almost everywhere in the fungal kingdom the number of molecular species appears to be much larger than what was earlier be recognized by conventional methods, see, for example, the fragmentation of *Aspergillus fumigatus* [41], *Candida parapsilosis* [42], or *Aureobasidium pullulans* [43]. Again, the dermatophytes seem to be exceptional. In the course of 150 years medical mycology mainly focusing on Caucasians in Europe, and with a wide diversity of diseases from different body parts, an exhaustive amount of pheno- and genotypes has been investigated in numerous publications. About 10 species can be categorized as common anthropophilic dermatophytes on the Eurasian and North-American continents. However, in the *Atlas of Clinical Fungi* [7], 103 basionyms, with 242 synonymous names in total, have been extracted from the literature to describe these same ≈10 species. It appears that the diversity seen with conventional approaches is much higher than the existing genetic diversity. We may conclude that the anthropophilic and perhaps also the zoophilic dermatophytes have been over-classified. Similar phenomena of over-classification are apparent in other fungal groups of practical importance and which have therefore been studied *in extenso*. For example, *Rhizopus* species are easy to grow in culture, and their culturing has started immediately after Pasteur’s time because of their role in fermentation processes of soy-based Asian foodstuffs. By 1920, 43 species were described in *Rhizopus microsporus* and *R. arrhizus*, which today are reduced to just two on molecular grounds [44, 45]. Another example is the ubiquitous saprobe *Alternaria alternata*, where the large number of morphological taxa mainly distinguished previously on the basis of conidial shape and three-dimensional conidiophore branching patterns were reduced to synonymy on the basis of genomic data [46].

### Phylogenetic Overview

It may be concluded that the taxonomy of common anthropophilic dermatophytes is now mature enough to be stabilized at the benefit of clinical routine. Taxa that are recognized today are not likely to be subject to drastic change in the near future. Trees do not suffer from taxon sampling effects, and nomenclatural stability is within reach. Additional species on the human host are to be expected only among rare taxa, such as *Trichophyton eriotrephon*, degenerate and difficult to identify species, such as *Microsporum aenygmaticum*, species from geographically remote areas, such as *Trichophyton concentricum*, or from coincidental infections of otherwise zoo- or geophilic species. Particularly, the geophilic dermatophytes have insufficiently been studied compared to their large number of potential host animals and environmental habitats, and in these groups a larger number of taxonomic novelties can be expected, which however have limited clinical relevance.

The current main genera *Epidermophyton, Microsporum*, and *Trichophyton* in their classical circumscription are based on morphology of macroconidia. This corresponds only partly with phylogeny in that species fulfilling the morphological criteria of *Trichophyton* partly cluster in derived anthropophilic clades, and partly in ancestral clades of prevalently geophilic species [24]. Consequently, a number of geophilic species which are phylogenetically remote from anthropophilic *Trichophyton* and hardly ever cause human infection are now included in routine identification panels [7]. From ecological and clinical viewpoints, the difference between the two groups is immense, because anthropophilic species are considered to be real pathogens in that they have evolutionary advantage of being transmitted between human hosts, whereas an overwhelming number of geophilic species are opportunistic and are acquired from a natural habitat in the environment. The combination of such highly diverse fungi in a single genus is not optimal and might lead to inefficient use of hospital resources when pathogenic species have to be distinguished from numerous non-human taxa. Molecular phylogeny using 5 genes clearly separated the preponderantly geophilic species from the remainder, comprising several zoophilic and a preponderantly anthropophilic clade, which confirms previously published topologies based on ITS [6], *TEF1* [28] and *CAL* [47]. Most zoophilic species compose clusters that are clearly separate from the preponderantly anthropophilic clades of *Trichophyton* and *Epidermophyton*. Now is the time to draw final conclusions and formulate the dermatophyte system in a modern sense, based on molecular phylogeny, supported by polyphasic data, and providing better tools for identification. This leads to a novel phylogenetic taxonomy and genus delimitation as outlined below. Main sets of criteria for species delimitation optimally should be based on the biological species concept, i.e., random mating with fertile progeny among members of the same species, and absence of mating between species. However, in microbiological practice, this criterion is often not easily applicable. Mating experiments and observation of fertile cleistothecia were particularly helpful to delineate species of the *M. gypseum* and of the *T. mentagrophytes* series [19, 20, 48–50]. However, sexual reproduction is often not known because the conditions under which teleomorphs are produced are unknown, or perhaps they may not exist at all. Inter-sterile populations may exist within what we regard as a single species. In dermatophytes, preponderance of a single mating type—which may have mating type-associated properties—may lead to asexual offshoots, explaining the clonal genetic composition of many species or other entities [51]. An alternative approach is genealogical concordance, i.e., the biological species concept expressed in silico. In the present study, this approach was adopted using four genes: LSU, ITS, 60S, and *TUB*. Different levels of resolution of clades were obtained with these genes. Listing the number of clades supported by bootstrap values >80 %, we observe ITS > *TUB* > 60S > LSU, yielding 44, 37, 32, and 17 clades, respectively (data not shown). For routine diagnostics, ITS is optimal, although for distinction of individual members of species complexes additional genes like *TUB* are necessary.

Once species have been delimited, the entities should be named according to the new rules of fungal nomenclature where Art. 59 of the ICBN regulating the pleomorphic naming system was abandoned. In principle the oldest name stands. From January 1, 2013, onwards, teleomorph names that are added later are considered as new combinations of the original basionym rather than as separate names. For older publications, the pleomorphic nomenclature still stand, in the sense that the different phases of the fungus are treated as facultative synonyms, even if they are introduced in the same paper and when based on the same type specimen. Often these types date back before 1958 since when explicit deposition was required (Art. 40 ICBN); in such cases the type of the teleomorph was selected as neotype of the species. In this way the currently accepted species is closely approached. The oldest, best known and widely used species names were mostly introduced even culture methods were available, and most of the nineteenth century names were based on clinical appearance only. Original materials are available of only a small selection of much younger taxa and synonyms. In order to maintain species names in current circumscriptions, widely used names are fixed by neotypes. In contrast, obsolete names for which no type materials are available are regarded as of doubtful identity and are thus permanently discarded.

## Nomenclature

Clades (A–G) in Fig. 3 are judged to represent genera. Table 2 summarizes and evaluates all genera described in dermatophyte taxonomy since 1841, and Table 3 provides the distribution of extant type species of each of these genera over the phylogenetic tree of Fig. 3. The oldest legitimate generic names available for each of the clades are valid, reducing later names as synonyms. The only exception is clade A for which the name *Trichophyton* is preferred over *Achorion*; a proposal for conservation of the former name is being prepared. Below the genera and species attributed to them are listed.

**Table 2:** List of generic names

***Achorion*** Remak, Diagnostische und pathogenetische Untersuchungen, in der Klinik des Herrn Geh. Raths Dr. Schonlein, B: 193, 1845. Type species: *A. schoenleinii* (Lebert) Remak ≡ *Trichophyton schoenleinii* (Lebert) Nannizzi (Clade 1)
***Aleurosporia*** Grigoraki, Annls Sci. Nat., Bot., Sér. 10, 7: 413, 1925. Type species: *A. acuminata* (Bodin) Grigoraki, Type material not known to be preserved; generic identity doubtful
***Arthroderma*** Berkeley, Outl. Brit. Fung. p. 357, 1860. Type species: *Arthroderma curreyi* Berkeley (Clade 6)
***Arthrosporia*** Grigoraki, Annls Sci. Nat., Bot., Sér. 10, 7: 414, 1925. Type species not indicated
***Bodinia*** Ota & Langeron, Annls Parasit. Hum. Comp. 1: 329, 1923. Type species: *B. violacea* (Sabouraud) Ota & Langeron ≡ *Trichophyton violaceum* Sabouraud (Clade 1)
***Chlamydoaleurosporia*** Grigoraki, C. R. Hebd. Séanc. Acad. Sci., Paris 179: 1425, 1924. Type species: *G. granulosa* (Sabouraud) Grigoraki ≡ *Trichophyton granulosum* Sabouraud (Clade 1)
***Chrysosporium*** Corda, *in* Sturm, Deutschl. Fl., 3 Abt. (Pilze Deutschl.) 3 (13): 85, 1833. Type species: *C. corii* Corda. Type species currently listed as *Chrysosporium merdarium* (Ehrenberg) Carmichael, a member of *Onygenaceae*
***Closteroaleurosporia*** Grigoraki, C. R. Hebd. Séanc. Acad. Sci., Paris 179: 1425, 1924. Type species: *C. audouinii* (Gruby) Grigoraki ≡ *Microsporum audouinii* (Clade 4)
***Closterosporia*** Grigoraki, Annls Sci. Nat., Bot., Sér. 10, 7: 415, 1925. Type species: *C. lanosa* (Sabouraud) Grigoraki ≡ *Microsporium lanosum* Sabouraud. Type material not known to be preserved; generic identity doubtful
***Ctenomyces*** Eidam, Beitr. Biol. Pfl. 3: 274, 1880. Type species: *C. serratus* Eidam [71, 73], an ancestral genus of *Arthrodermataceae* and a younger synonym of *Arthroderma*
***Ectotrichophyton*** Castellani & Chalmers, Man. Trop. Med., 3rd ed. p. 1002, 1919. Type species: *E. mentagrophytes* (Robin) Castellani & Chalmers ≡ *Trichophyton mentagrophytes* (Robin) Blanchard (Clade 6)
***Ectotrichophyton*** subgen. ***Microtrichophyton*** Castellani & Chalmers, Man. Trop. Med., 3rd ed. p. 1004, 1919 ≡ *Microtrichophyton* (Castellani & Chalmers) Neveu-Lemaire, Précis Parasitol. Hum., ed. 2: 46, 1921. Type species *M. felineum* (Blanchard) Neveu-Lemaire ≡ *Trichophyton felineum* Blanchard. Possibly *Myceliophthora vellerea* (Sacc. & Speg.) v. Oorschot was concerned, but type material not known to be preserved; generic identity doubtful
***Endodermophyton*** Castellani & Chalmers, Man. Trop. Med. p. 610, 1910. Type species: *E. castellanii* (Perry) Castellani & Chalmers ≡ *Trichophyton castellanii* Perry. Type material not known to be preserved; generic identity doubtful
***Epidermomyces*** Loeffler, Mykosen 26: 446, 1983. Type species: *E. floccosum* (Harz) Loeffler ≡ *Acrothecium floccosum* Harz ≡ *Epidermophyton floccosum* (Harz) Langeron & Milochevitch (Clade 2)
***Epidermophyton*** E. Lang, Vierteljahresschr. Dermatol. Syph. 11: 263, 1879. Rejected name, Art. 14.7 ICBN [72]
*Epidermophyton* Megnin, C. R. Soc. Biol., Paris 33: 405, 1881. Rejected name, Art. 14.7 ICBN [72]
***Epidermophyton*** Sabouraud, Arch. Méd. Exp. Anat. Path. 19: 754, 1907. Type species: *E. inguinale* Sabouraud, Arch. Méd. Exp. Anat. Path. 19: 565, 1907. Type lost. The generic name was conserved (Art. 14.7 ICBN) [72] with *Epidermophyton floccosum* (Harz) Langeron & Milochevitch as type species (Clade 2)
***Favomicrosporon*** Benedek, Mycopath. Mycol. Appl. 31: 111, 1967. Type species: *F. pinettii* Benedek, Mycopath. Mycol. Appl. 31: 111, 1967 = *Microsporum fulvum* (Clade 3)
***Favotrichophyton*** Neveu-Lemaire, Précis Parasitol. Hum., ed. 2: 55, 1921. Type species: *F. ochraceum* (Sabouraud) Neveu-Lemaire ≡ *Trichophyton ochraceum* Sabouraud. In literature treated as synonym of *T. verrucosum* Bodin, but type material not known to be preserved; generic identity doubtful
***Grubyella*** Ota & Langeron, Annls Parasit. Hum. Comp. 1: 330, 1923. Type species: *G. schoenleinii* (Lebert) Ota & Langeron ≡ *Trichophyton schoenleinii* (Lebert) Nannizzi (Clade 1).
***Kaufmannwolfia*** Galgoczy & Novák, *in* Bakacs, Azágos Orszö Kozegészségügyi Intézet Müködése: 225, 1962. Type species: *K. interdigitalis* (Priestley) Galgoczy & Novak ≡ *Trichophyton interdigitale* Priestley (Clade 1)
***Keratinomyces*** Vanbreuseghem, Bull. Acad. R. Sci. Belg., Cl. Sci., Sér. 5, 38: 1075, 1952. Type species: *K. ajelloi* Vanbreuseghem = anamorphic *Arthroderma uncinatum* Dawson & Gentles (Clade 6)
***Langeronia*** Vanbreuseghem, Annls Parasit. Hum. Comp. 25: 506, 1950. Type species: *L. soudanensis* (Joyeux) Vanbreuseghem ≡ *Trichophyton soudanense* Joyeux = *Trichophyton rubrum* (Clade 1)
***Langeronites*** Ansel (1957). Type species: *Langeronites persicolor* (Sabouraud) Ansel, 1957 ≡ *Nannizzia persicolor* (Sabouraud) Stockdale (Clade 3). No description of this genus could be recovered
***Lepidophyton*** Tribondeau, Arch. Méd. Navale 72: 48, 1899. No species listed; invalid genus
***Lophophyton*** Matruchot & Dassonville, Rev. Gén. Bot. 11: 432, 1899. Type species: *L. gallinae* Matruchot & Dassonville ≡ *Microsporum gallinae* (Mégnin) Grigoraki (Clade 5)
***Megatrichophyton*** Neveu-Lemaire, Précis Parasitol. Hum., ed. 2, p. 46, 1921. Type species *M. equinum* (Gedoelst) Neveu-Lemaire ≡ *Trichophyton equinum* Gedoelst (Clade 1)
***Microides*** De Vroey, Ann. Soc. Belg. Méd. Trop. 50: 24, 1970. Type species not indicated; genus invalid according to ICBN Art. 40.1
***Microsporum*** Gruby, C. R. Hebd. Séanc. Acad. Sci., Paris 17: 301, 1843. Type species: *M. audouinii* Gruby (Clade 4)
***Nannizzia*** Stockdale, Sabouraudia 1: 45, 1961. Type species: *N. incurvata* Stockdale (Clade 3)
***Neotrichophyton*** Castellani & Chalmers, Man. Trop. Med., 3rd ed. p. 1001, 1919. Type species: *N. flavum* (Bodin) Castellani & Chalmers ≡ *Trichophyton flavum* Bodin, Champignons Paras. Homme Anim. Domest. p. 119, 1902. Type material not known to be preserved; generic identity doubtful
***Pinoyella*** Castellani & Chalmers, Man. Trop. Med., 3rd ed. p. 1023, 1919. Type species: *P. simii* (Pinoy) Castellani & Chalmers ≡ *Epidermophyton simii* Pinoy ≡ *Trichophyton simii* (Clade 1)
***Sabouraudites*** Ota & Langeron, Annls Parasit. Hum. Comp. 1: 326, 1923. Type species*: S. asteroides* (Sabouraud) Ota & Langeron ≡ *Trichophyton asteroides* Sabouraud, Malad. Cuir Chev. 3: 347, 1910. Type material not known to be preserved; generic identity doubtful [7]
***Sabouraudiella*** Boedijn, Mycopath. Mycol. Appl. 6: 123, 1953. Type species: *S. purpureum* (Bang) Boedijn ≡ *Trichophyton purpureum* Bang, Annals Derm. Syph. 5, Sér. 1: 238, 1910 = *Trichophyton rubrum* (Clade 1)
***Schoenleinium*** Johan-Olsen, Zentbl. Bakt. ParasitKde, Abt. II, 3: 276, 1897. Type species: *S. achorion* Johan-Olsen. Type material not known to be preserved; generic identity doubtful
***Shanorella*** Benjamin, Aliso 3: 319, 1956. Type species: *S. spirotricha* Benjamin, classifies outside *Arthrodermataceae*, probably member of *Onygenaceae*
***Spiralia*** Grigoraki, Annls Sci. Nat., Bot., Sér. 10, 7: 409, 1925. Nom. illegit., Art. 53.1, non *Spiralia* Toula 1900 (fossil Algae). Type species: *S. asteroides* (Sabouraud) Grigoraki ≡ *Trichophyton asteroides* Sabouraud. Type material not known to be preserved; generic identity doubtful [7]
***Thallomicrosporon*** Benedek, Mycopath. Mycol. Appl. 23: 96, 1964. Type species: *T. kuehnii* Benedek, Mycopath. Mycol. Appl. 23: 96, 1964. Type material not known to be preserved; generic identity doubtful
***Trichomyces*** Malmsten, Arch. Anat. Physiol. Wiss. Med., 1848: 14, 1848. Type species: *T. tonsurans* (Malmsten) Guéguen, Bull. Calif. Acad. Sci.: 14, 1848 ≡ *Trichophyton tonsurans* Malmsten (Clade 1). This name and reference are listed in various databases; probably a misspelling for *Trichophyton* is concerned
***Trichophyton*** Malmsten, Arch. Anat. Physiol. Wiss. Med. 1848: 14, 1848. Type species: *T. tonsurans* Malmsten (Clade 1)
***Veronaia*** Benedek, Mycopath. Mycol. Appl. 14: 115, 1961. Type species: *V. castellanii* Benedek. Type material not known to be preserved; generic identity doubtful

**Table 3:** Confirmed generic synonymies

Clade 1: *Achorion* 1845 = *Trichophyton* 1848 = *Trichomyces* 1848 = *Ectotrichophyton* 1919 = *Pinoyella* 1919 = *Megatrichophyton* 1921 = *Grubyella* 1923 = *Bodinia* 1923 = *Langeronia* 1950 = *Sabouraudiella* 1951 = *Kaufmannwolfia* 1962
Clade 2: *Epidermophyton* 1907 = *Epidermomyces* 1983
Clade 3: *Langeronites* 1957 (nom. inval.) = *Nannizzia* 1961 = *Favomicrosporon* 1967
Clade 4: *Microsporum* 1843 = *Closteroaleurosporia* 1924
Clade 5: *Lophophyton* 1899
Clade 6: *Arthroderma* 1860 = *Keratinomyces* 1962 = *Ctenomyces* 1880

### Clade A: *Trichophyton*

Colonies mostly cottony, white to yellowish, with a cream-colored, brown, red, violet colony reverse. Hyphae thin-walled, hyaline. Thallic macroconidia and microconidia, if present, terminally on or alongside undifferentiated hyphae. Macroconidia, 2- or multi-celled, thin- and smooth-walled, hyaline, cylindrical, or clavate to cigar-shaped. Microconidia thin- and smooth-walled, hyaline, 1-celled, ovoidal, pyriform to clavate. Sexual state sometimes present after mating, arthroderma-like.

Type species: *Trichophyton tonsurans* Malmsten.

**1.** ***Trichophyton benhamiae*** (Ajello & Cheng) Gräser & de Hoog, **comb. nov.**

Basionym: *Arthroderma benhamiae* Ajello & Cheng, Sabouraudia 5: 232, 1967. Holotype NCDC B765d; type strain cross of CBS 623.66 = ATCC 16781 = CDC X797 (MT+) × CBS 624.66 = ATCC 16782 = CDC X798 (MT−), UK, L. Ajello. Zoophilic species, mainly on guinea pigs [52], occasionally other animals [53]. A white and a yellow phenotype are known, the yellow genotype containing MT− strains only [20]; a hybridization depression is noted with the remaining lineages. Contet-Andonneau & Leyer [54] invalidly introduced *Trichophyton erinacei* var. *porcellae* (without indication of type specimen, Art. 52 ICBN) matching the yellow phenotype. Note that with multilocus sequencing the mating types deviate slightly from remaining strains (Fig. 4).

**2.** ***Trichophyton bullosum*** Lebasque, Champ. Teign. Cheval Bovidés p. 53, 1933. Type strain: CBS 363.35, from horse, France. Zoophilic species [55].

**3.** ***Trichophyton concentricum*** Blanchard, *in* Bouchard, Traité Path. Gén. 2: 916, 1896 ≡ *Lepidophyton concentricum* (Blanchard) Gedoelst, Champ. Paras. Homme Anim. Domest. p. 147, 1902 ≡ *Aspergillus concentricum* (Blanchard) Castellani, Trans. Int. Derm. Congr. 6: 671, 1907 ≡ *Endodermophyton concentricum* (Blanchard) Castellani & Chalmers, Man. Trop. Med. p. 610, 1910 ≡ *Oospora concentrica* (Blanchard) Hanawa & Nagai, Jpn. J. Derm. Urol., Suppl., p. 47, 1917 ≡ *Achorion concentricum* (Blanchard) Guiart & Grigoraki, Lyon Méd. 141: 377, 1928 ≡ *Mycoderma concentricum* (Blanchard) Vuillemin, C. R. Hebd. Séanc. Acad. Sci., Paris 89: 405, 1929. Neotype strain: CBS 196.26 = IFO 5926, A. Castellani, 1926. Anthropophilic species causing tinea imbricata in Polynesia [56].

**4.** ***Trichophyton equinum*** Gedoelst, Champ. Paras. Homme p. 88, 1902 ≡ *Ectotrichophyton equinum* (Gedoelst) Castellani & Chalmers, Man. Trop. Med., ed. 3, p. 1007, 1919 ≡ *Megatrichophyton equinum* (Gedoelst) Neveu-Lemaire, Précis Parasitol. Hum., ed. 5, p. 54, 1921 ≡ *Ctenomyces equinus* (Gedoelst) Nannizzi, Tratt. Micopat. Um. 4: 144, 1934. **Neotype designated here:** CBS 270.66, from horse, USA, L.K. George. Zoophilic species, but, at least based on DNA sequences, doubtfully distinct from *T. tonsurans* which is generally regarded as anthropophilic. The species are phenotypically distinguished by brown colonies and larger microconidia in *T. tonsurans*.

Fac. syn.: *Trichophyton areolatum* Negroni, Annls Parasit. Hum. Comp. 7: 438, 1929. Type strain: CBS 285.30, Argentina, P. Negroni.

Fac. syn.: *Trichophyton equinum* Gedoelst var. *autotrophicum* J.M.B. Smith, Jolly, Georg & Connole, Sabouraudia 6: 297, 1968. Type strain: CBS 100080 = ATCC 22443 = IMI 133568, from horse, New Zealand.

**5.** ***Trichophyton eriotrephon*** Papegaaij, Nederl. Tijdschr. Geneesk. 69: 885, 1925. Type strain: CBS 220.25, from ringworm of female patient, The Netherlands, J. Papegaaij, 1925.

**6.** ***Trichophyton erinacei*** (J.M.B. Smith & Marbles) Quaife; *Trichophyton mentagrophytes* (Robin) Blanchard var. *erinacei* J.M.B. Smith & Marbles, Sabouraudia 3: 9, 1963 ≡ *Trichophyton erinacei* (J.M.B. Smith & Marbles) Quaife, J. Clin. Path. 19: 178, 1966 ≡ *Arthroderma benhamiae* Ajello & Cheng var. *erinacei* (J.M.B. Smith & Marbles) Takashio, Bull. Soc. Fr. Mycol. Méd. 4: 47, 1975. Holotype: IMI 101051; type strain: CBS 511.73 = ATCC 28443 = IMI 101051 = NCPF 375, from hedgehog, New Zealand.

**7.** ***Trichophyton interdigitale*** Priestley, Med. J. Aust. 4: 475, 1917 ≡ *Sabouraudites interdigitalis* (Priestley) Ota & Langeron, Annls Parasit. Hum. Comp. 1: 328, 1923 ≡ *Epidermophyton interdigitale* (Priestley) MacCarthy, Archs Derm. Syph. 6: 24, 1925 ≡ *Trichophyton mentagrophytes* (Robin) Blanchard var. *interdigitale* (Priestley) Moraes, Anais Bras. Derm. Sif. 25: 230, 1950 ≡ *Kaufmannwolfia interdigitalis* (Priestley) Galgóczy & Novák, *in* Bakács, Az Orsz. Köz. Intéz. Mük. p. 224, 1962 ≡ *Microides interdigitalis* (Priestley) De Vroey, Ann. Soc. Belg. Méd. Trop. 50: 25, 1970. Neotype strain: CBS 428.63, from human foot, The Netherlands, M. Bruining [6]. Anthropophilic species, almost exclusively found in non-inflammatory tinea unguium and tinea pedis. The species may be regarded as a clonal offshoot of *T. mentagrophytes*. The position of CBS 119447 requires further study.

Fac. syn.: *Trichophyton batonrougei* Castellani, J. Trop. Med. Hyg. 42: 373, 1939 ≡ *Trichophyton mentagrophytes* (Robin) Blanchard var. *batonrougei* (Castellani) de Vries & Cormane, Ned. Tijdschr. Geneeskd. 109: 1426, 1965. Type strain: CBS 425.63, A. Castellani.

Fac. syn.: *Trichophyton candelabrum* Listemann, Castellania 1: 53, 1973. Type strain: CBS 647.73, from human toenail, Germany, H. Listemann.

Fac. syn.: *Trichophyton krajdenii* J. Kane, J.A. Scott & Summerbell, Mycotaxon 45: 309, 1992. Type: CBS 475.93 = UAMH 3244, from human skin, Canada, J. Kane.

Fac. syn.: *Trichophyton radicosum* Catanei, Arch. Inst. Pasteur Algér. 15: 267, 1937. Type strain: CBS 304.38, A. Catanei, May 1938. Note that in the multilocus tree (Fig. 4), the position of the type strain is unresolved.

**8.** ***Trichophyton mentagrophytes*** (Robin) Blanchard; *Microsporum mentagrophytes* Robin, Hist. Nat. Vég. Paras. Homme Anim. p. 430, 1853 ≡ *Sporotrichum mentagrophytes* (Robin) Saccardo, Syll. Fung. 4: 100, 1886 ≡ *Trichophyton mentagrophytes* (Robin) Blanchard, Traité Pathol. Gén. 2: 811, 1896 ≡ *Ectotrichophyton mentagrophytes* (Robin) Castellani & Chalmers, Man. Trop. Med., ed. 3. p. 1005, 1919 ≡ *Ctenomyces mentagrophytes* (Robin) Langeron & Milochevitch, Annls Parasit. Hum. Comp. 8: 484, 1930 ≡ *Spiralia mentagrophytes* (Robin) Grigoraki, C. R. Séanc. Soc. Biol. 109: 186, 1932 ≡ *Microides mentagrophytes* (Robin) De Vroey, Ann. Soc. Belg. Méd. Trop. 50: 25, 1970. As neotype, CBS 318.56 has been selected [6], but this was disputed by several authors [57–59]. Chollet et al. [60] convincingly showed that the original case of C. Robin concerned a human tinea barbae, a disorder generally ascribed to zoophilic species. Isolates of this species show some ITS diversity but are either from animals or from patients with inflammatory dermatophytoses indicating an animal origin; reservoirs are hunting cats, dogs [52], mice [19] and horses [61]. Isolates are able to mate with *Arthroderma* strains [50]. An alternative **neotype designated herewith** IHEM 4268, from tinea corporis of human face, Brussels, Belgium, which is more in accordance with the protologue. Note that until recently a distinction was made between anthropophilic and zoophilic strains of *T. mentagrophytes* [62]. Truly anthropophilic, low-inflammatory strains correspond with the clonal offshoot *T. interdigitale*, while more inflammatory human infections by zoophilic strains match with *T. mentagrophytes s. str*.

Fac. syn.: *Bodinia abyssinica* Agostini, Atti Ist. Bot. Lab. Crittogam. Univ. Pavia, Ser. 4, 2: 123, 1931 ≡ *Trichophyton abyssinicum* (Agostini) Nannizzi, Tratt Micopat. Um. 4: 174, 1934 ≡ *Favotrichophyton abyssinicum* (Agostini) C.W. Dodge, Med. Mycol. p. 517, 1935. Type strain: CBS 126.34, from human skin, G. Pollacci, 1934.

Fac. syn.: *Arthroderma vanbreuseghemii* Takashio, Ann. Soc. Belg. Méd. Trop. 53: 547, 1973. Type strain: CBS 646.73 = ATCC 28145 = CECT 2900 = IHEM 3299 = NCPF 452 (MT+), M. Takashio.

**9.** ***Trichophyton quinckeanum*** (Zopf) MacLeod & Münde; *Oidium quinckeanum* Zopf, Die Pilze p. 481, 1890 ≡ *Achorion quinckeanum* (Zopf) Bodin, Archs Parasit. 5: 5–30, 1902 ≡ *Sabouraudites quinckeanus* (Zopf) Ota & Langeron, Annls Parasit. Hum. Comp. 1: 328, 1923 ≡ *Closteroaleuriospora quinckeana* (Zopf) Grigorakis, Annls Sci. Nat., Bot., Sér. 10, 12: 412, 1925 ≡ *Microsporum quinckeanum* (Zopf) Guiart & Grigorakis, Lyon Méd. 141: 377, 1928 ≡ *Trichophyton quinckeanum* (Zopf) MacLeod & Münde, Pract. Handb. Skin p. 361, 1940 ≡ *Trichophyton gypseum* Bodin var. *quinckeanum* (Zopf) Frágner, Česká Mykol. 10: 106, 1956 ≡ *Trichophyton mentagrophytes* (Robin) Blanchard var. *quinckeanum* (Zopf) J.M.B. Smith & Austwick, *in* Cotchin & Roe, Path. Lab. Rats Mice p. 684, 1967. **Neotype designated herewith:** IHEM 13697 = RV 32626 = CDC X393, from mouse favus, Philadelphia, USA, H. Blank. Zoophilic species causing favus on mice [59]. Member of the *T. mentagrophytes* series.

**10.** ***Trichophyton rubrum*** (Castellani) Semon; *Epidermophyton rubrum* Castellani, Phil. J. Sci. 5: 203, 1910 ≡ *Trichophyton rubrum* (Castellani) Semon, Br. J. Derm. Syph. 34: 398, 1922 ≡ *Sabouraudites ruber* Ota & Langeron, Annls Parasit. Hum. Comp. 1: 328, 1923 ≡ *Sabouraudiella rubra* (Castellani) Boedijn, Mycopath. Mycol. Appl. 6: 125, 1951. Neotype strain: CBS 392.58, from human, The Netherlands, H. Esseveld. Anthropophilic species, the most prevalent recognized infectious agent in onychomycoses (tinea unguium) and tinea pedis, also causing tinea cruris and tinea corporis; it has a global distribution. *Trichophyton megninii* Blanchard is often listed as a synonym of *Trichophyton rubrum*, but no type material is known to exist.

Fac. syn.: *Trichophyton balcaneum* Castellani, J. Trop. Med. Hyg. 22: 174, 1919. Type strain: CBS 359.62, from human, USA, T. Benedek. The identity of this strain is uncertain and should be re-investigated.

Fac. syn.: *Trichophyton rodhainii* Vanbreuseghem, Annls Parasit. Hum. Comp. 24: 244, 1949 *Trichophyton rubrum* Castellani var. *rodhainii* (Vanbreuseghem) Armijo & Lachapelle, Annls Derm. Vénéréol. 108: 990, 1981. Type strain: CBS 376.49, from tinea cruris of Caucasian in Congo, R. Vanbreuseghem.

Fac. syn.: *Trichophyton fluviomuniense* Pereiro Miguens, Sabouraudia 6: 315, 1968. Type strain: CBS 592.68 = ATCC 22402, from human skin, Guinea, M. Pereiro Miguens.

Fac. syn.: *Trichophyton fischeri* Kane, Sabouraudia 15: 239, 1977. Type strain: CBS 100081 = ATCC 32871 = IMI 213848, culture contaminant, Toronto, Canada.

Fac. syn.: *Trichophyton raubitschekii* Kane, Salkin, Weitzman & Smitka, Mycotaxon 13: 260, 1981 ≡ *Trichophyton rubrum* (Castellani) Semon var. *raubitschekii* (Kane, Salkin, Weitzman & Smitka) Brasch, Mycoses 50, Suppl. 2: 2, 2007. Type strain: CBS 100084 = ATCC 42631, from human, Canada, J. Kane.

Fac. syn.: *Trichophyton kanei* Summerbell, Mycotaxon 28: 511, 1987. Type strain: CBS 289.86 = ATCC 62345 = TRTC 50887, from human skin, Canada, R.C. Summerbell.

**11.** ***Trichophyton schoenleinii*** (Lebert) Nannizzi; *Oidium schoenleinii* Lebert, Physiol. Path. 2: 490, 1845 ≡ *Achorion schoenleinii* (Lebert) Remak, Diagn. Pathog. Unters. p. 13, 1845 ≡ *Schoenleinium achorion* Johan-Olsen, Zentbl. Bakt. Parasitkde, Abt. 2, 3: 276, 1897 (name change) ≡ *Grubyella schoenleinii* (Lebert) Ota & Langeron, Annls Parasit. Hum. Comp. 1: 320, 1923 ≡ *Arthrosporia schoenleinii* (Lebert) Grigoraki, Annls Sci. Nat., Bot., Sér. 7: 414, 1925 ≡ *Sporotrichum schoenleinii* (Lebert) Saccardo, *in* Vuillemin, Champ. Paras. Myc. Homme p. 69, 1931 ≡ *Trichophyton schoenleinii* (Lebert) Nannizzi, Tratt. Micopat. Um. 4: 198, 1934. **Neotype designated herewith:** CBS 458.59, from human scalp, The Netherlands, F.H. Oswald. Anthropophilic species.

**12.** ***Trichophyton simii*** (Pinoy) Stockdale, MacKenzie & Austwick; *Epidermophyton simii* Pinoy, C. R. Soc. Biol. 72: 59, 1912 ≡ *Pinoyella simii* (Pinoy) Castellani & Chalmers, Man. Trop. Med., ed. 3, p. 1023, 1919 ≡ *Arthroderma simii* (Pinoy) Stockdale, MacKenzie & Austwick, Sabouraudia 4: 113, 1965 ≡ *Trichophyton simii* (Pinoy) Stockdale, MacKenzie & Austwick, Sabouraudia 4: 114, 1965. The type material of *E. simii* is not known to be preserved. A teleomorph was introduced by Stockdale et al. [63], which is here taken as a new combination and is considered to be representative for the species. Holotype IMI 98944, authentic strains: CBS 417.65 = ATCC 16448 = IHEM 4420 = IMI 101695 = NCPF 394 (MT−), CBS 448.65 = ATCC 16447 = IHEM 4421 = IMI 101693 = NCPF 494 (MT+), CBS 449.65 = IMI 101694 = NCPF 393 (MT+), all from poultry, India, C.O. Dawson. Zoophilic species.

**13.** ***Trichophyton soudanense*** Joyeux, C. R. Seanc. Soc. Biol. 73: 15, 1912 ≡ *Langeronia soudanensis* (Joyeux) Vanbreuseghem, Ann. Soc. Belg. Méd. Trop. 30: 888, 1950. **Neotype designated herewith:** IHEM 19751 = RV 44663, from tinea capitis, Lomé, Togo, Tchalim, 1988. Anthropophilic species, very close to, perhaps even indistinguishable from *T. violaceum*, both species causing tinea capitis in northern Africa. More detailed studies are needed to establish species borderlines.

**14.** ***Trichophyton tonsurans*** Malmsten, harskärende Mögel. Bidrag till utredande af de sjukdomar, som valla harets affall. Stockholm, gr. 8, 1845; Arch. Anat. Physiol. Wiss. Med. 1848: 14, 1848 ≡ *Trichomyces tonsurans* (Malmsten) Malmsten, Arch. Anat. Physiol. Wiss. Med. 1848: 14, 1848 ≡ *Oidium tonsurans* (Malmsten) Zopf, Die Pilze p. 482, 1890. Neotype strain: CBS 496.48, from human scalp, France, M. Rivalier. Anthropophilic species [6].

Fac. syn.: *Trichophyton floriforme* Beintema, Arch. Dermatol. 169: 575, 1934. Type strain: CBS 318.31, K. Beintema.

Fac. syn.: *Trichophyton immergens* Milochevitch, C. R. Hebd. Séanc. Acad. Sci., Paris 124: 469, 1937. Type strain: CBS 338.37, from human glabrous skin, Serbia, S. Milochevitch.

**15.** ***Trichophyton verrucosum*** Bodin, Champ. Paras. Homme p. 121, 1902 ≡ *Ectotrichophyton verrucosum* (Bodin) Castellani & Chalmers, Man. Trop. Med., ed. 3, p. 1003, 1919 ≡ *Favotrichophyton verrucosum* (Bodin) Neveu-Lemaire, Précis Parasitol. Hum., ed. 5, p. 55, 1921. **Neotype designated herewith:** CBS 365.53, from cow, F. Blank. Zoophilic species on cattle.

**16.** ***Trichophyton violaceum*** Sabouraud, *in* Bodin, Champ. Paras. Homme p. 113, 1902 ≡ *Achorion violaceum* (Sabouraud) Bloch, Derm. 18: 815, 1911 ≡ *Sabouraudites violaceum* (Sabouraud) Ota & Langeron, Annls Parasit. Hum. Comp. 1: 328, 1923 ≡ *Bodinia violacea* (Sabouraud) Ota & Langeron, Annls Parasit. Hum. Comp. 1: 329, 1923 ≡ *Arthrosporia violacea* (Sabouraud) Grigoraki, Annls Sci. Nat., Bot., Sér. 10, 7: 414, 1925 ≡ *Favotrichophyton violaceum* (Sabouraud) C.W. Dodge, Med. Mycol. p. 523, 1935. Neotype strain: CBS 374.92, from human, The Netherlands, C.S. Tan [24]. Anthropophilic species. Note that molecularly the species cannot be distinguished from *T. rubrum* (Fig. 4). Strains from human scalp generate *T. violaceum* phenotypes, so probably mutations in the pentaketide pathway interfering with the production of pigmented secondary metabolites are concerned.

Fac. syn.: *Trichophyton yaoundei* Cochet & Doby-Dubois, Annls Parasit. Hum. Comp. 32: 585, 1957. Type strain: CBS 305.60, G. Cochet, November 1960.

Fac. syn.: *Trichophyton kuryangei* Vanbreuseghem & Rosenthal, Annls Parasit. Hum. Comp. 36: 802, 1961. Type strain: CBS 517.63 = RV 8289, from tinea capitis of black infant, Kuryange, Usumbura Province, Ruanda Burundi, R. Vanbreuseghem.

### Clade B: *Epidermophyton*

Colonies cottony, white to yellowish, with a cream-colored or brownish colony reverse. Hyphae thin-walled, hyaline. Thallic macroconidia terminally on or alongside undifferentiated hyphae, multi-celled, thin- and smooth- or rough-walled, hyaline, cigar-shaped. Microconidia absent. Sexual state unknown.

Type species: *Acrothecium floccosum* Harz.

**1.** ***Epidermophyton floccosum*** (Harz) Langeron & Milochevitch; *Acrothecium floccosum* Harz, Bull. Soc. Imp. Nat. Moscou 44: 124, 1871 ≡ *Blastotrichum floccosum* (Harz) Belese & Voglino, Add. Syll. Nr. 3604, 1886 ≡ *Dactylium floccosum* (Harz) Sartory, Champ. Paras. Homme Anim. p. 871, 1923 ≡ *Epidermophyton floccosum* (Harz) Langeron & Milochevitch, Annls Parasit. Hum. Comp. 8: 495, 1930 ≡ *Epidermomyces floccosus* (Harz) Loeffler, Mykosen 26: 446, 1983. **Neotype designated herewith:** CBS 230.76, from human, R.A. Zappey. Anthropophilic species.

### Clade C: *Nannizzia*

Colonies mostly cottony to powdery, whitish to brown, with a cream-colored, brown or red. Hyphae thin-walled, hyaline. Thallic macroconidia and microconidia, if present, arranged in orthotropically arranged hyphal systems. Macroconidia, 2- or multi-celled, thin- and smooth- or rough-walled, hyaline, cylindrical, or clavate to cigar-shaped. Microconidia thin- and smooth-walled, hyaline, 1-celled, ovoidal, pyriform to clavate. Sexual state commonly present after mating, arthroderma-like.

Type species: *Nannizzia incurvata* Stockdale.

**1.** ***Nannizzia aenygmaticum*** (Hubka, Dobiášová & Kolařík) Gräser & de Hoog, **comb. nov.**

Basionym: *Microsporum aenygmaticum* Hubka, Dobiášová & Kolařík, Med. Mycol. 52: 389, 2014. Holotype: PRM 922698, type strain: CBS 134549 = CCF4608, skin of 46-year-old female, Czech Republic, Ostrava, S. Dobiášová.

**2.** ***Nannizzia corniculata*** (Takashio & De Vroey) Gräser & de Hoog, **comb. nov.**

Basionym: *Nannizzia corniculata* Takashio & De Vroey, Mycotaxon 14: 384, 1982 ≡ *Arthroderma corniculatum* (Takashio & De Vroey) Weitzman, McGinnis, Padhye & Ajello, Mycotaxon 25: 513, 1986. Holotype: CBS-H 7400; type culture: CBS 364.81 = ATCC 46541 = IHEM 4409, from soil, Somalia.

**3** ***. Nannizzia duboisii*** (Vanbreuseghem) Gräser & de Hoog, **comb. nov.**

Basionym: *Sabouraudites duboisii* Vanbreuseghem, Annls Parasit. Hum. Comp. 24: 254, 1949 ≡ *Microsporum duboisii* (Vanbreuseghem) Ciferri, Man. Mic. Med., ed. 2: 414, 1960. Type strain: CBS 349.49, from human, Zaire, R. Vanbreuseghem.

**4.** ***Nannizzia fulva*** (Uriburu) Stockdale; *Microsporum fulvum* Uriburu, Argent. Med. 7, 1909 ≡ *Sabouraudites fulvus* (Uriburu) Ota & Langeron, Annls Parasit. Hum. Comp. 1: 329, 1923 ≡ *Closterosporia fulva* (Uriburu) Grigoraki, Annls. Sci. Nat., Bot. Sér. 10, 7: 411, 1925 ≡ *Nannizzia fulva* (Uriburu) Stockdale, Sabouraudia 3: 120, 1963 ≡ *Nannizzia gypsea* (Uriburu) Stockdale var. *fulva* (Uriburu) Apinis, Mycol. Pap. 96: 33, 1964 ≡ *Arthroderma fulvum* (Uriburu) Weitzman, McGinnis, Padhye & Ajello, Mycotaxon 25: 513, 1986. Holotype: IMI 10065, type strain: CBS 287.55, from human, Argentina, E. Rivalier. Types of teleomorph: CBS 168.64 = ATCC 16446 = IHEM 3296 = IMI 086180 = NCPF 391 (MT−) × ATCC 16445 = IHEM 3292 = IMI 086179 = NCPF 390 (MT+), both from soil, Hungary, S. Szathmary. Geophilic species.

Fac. syn.: *Keratinomyces longifusus* Flórián & Galgóczy, Mycopath. Mycol. Appl. 24: 76, 1964. Type strain: CBS 243.64 = ATCC 22397, from human, Hungary, E. Flórián, May 1964.

Fac. syn.: *Microsporum boullardii* Dominik & Majchrowicz, Ekol. Polska, Ser. A, 13: 426, 1965. Type strain: CBS 599.66 = ATCC 22399, from soil, Guinea, T. Dominik.

Fac. syn.: *Favomicrosporon pinettii* Benedek, Mycopath. Mycol. Appl. 31: 111, 1967. Authentic strains: CBS 146.66 = ATCC 16482, CBS 147.66 = ATCC 16481, T. Benedek.

Fac. syn.: *Microsporum ripariae* Hubálek & Rush-Munro, Sabouraudia 11: 288, 1973. Type strain: CBS 529.71 = ATCC 28005, from sand martin swallow (*Riparia riparia*), Czechia, Z. Hubálek.

**5.** ***Nannizzia gypsea*** (Nannizzi) Stockdale; *Gymnoascus gypseus* Nannizzi, Atti Accad. Fisioscr. Siena Med.-Fis. 2: 93, 1927 [*non Trichophyton gypseum* Bodin, Champ. Paras. Homme p. 115, 1902] ≡ *Nannizzia gypsea* (Nannizzi) Stockdale, Sabouraudia 3: 119, 1964 ≡ *Arthroderma gypseum* (Nannizzi) Weitzman, McGinnis, Padhye & Ajello, Mycotaxon 25: 514, 1986. Neotype strain: CBS 258.61 = IMI 80558, from soil, Australia, D.M. Griffin, Nov. 1961. Geophilic species.

Fac. syn.: *Microsporum appendiculatum* Bhat & Miriam, *in* Miriam & Bhat, Kavaka 25: 93, 1998. Holotype: GUFH 010, India, Goa University campus, herbarium specimen on decomposing goat dung, J. Miriam, 1996. Sharma et al. [64] showed that strains with appendiculate conidia were genetically identical to *M. gypseum*.

Fac. syn.: *Microsporum gypseum* (Bodin) Guiart & Grigoraki var. *vinosum* Gordon & Lusick, Archs Derm. 91: 562, 1965. Type strain: CBS 100.64 = ATCC 16428, from human, USA, 1964, M.A. Gordon.

**6.** ***Nannizzia incurvata*** Stockdale, Sabouraudia 1: 46, 1961 ≡ *Nannizzia gypsea* (Nannizzi) Stockdale var. *incurvata* (Stockdale) Apinis, Mycol. Pap. 96: 32, 1964 ≡ *Arthroderma incurvatum* (Stockdale) Weitzman, McGinnis, Padhye & Ajello, Mycotaxon 25: 514, 1986 ≡ *Microsporum incurvatum* (Stockdale) P.-L. Sun & Y.-M. Ju, Med. Mycol. 52: 280, 2014. Holotype: dried culture IMI 82777, type strain: CBS 174.64 = IMI 82777 = NCPF 236, from human skin, UK, P.M. Stockdale. Geophilic species, although also human infections occur. Stockdale [11] reported production of ascocarps using human-derived strains only.

**7.** ***Nannizzia nana*** (Fuentes) Gräser & de Hoog, **comb. nov.**

Basionym: *Microsporum gypseum* (Bodin) Guiart & Grigoraki var. *nanum* Fuentes, Aboulafia & Vidal, J. Invest. Derm. 23: 56, 1954 (invalid) ≡ *Microsporum nanum* Fuentes, Aboulafia & Vidal ex Fuentes, Mycologia 48: 614, 1956. Type strain: CBS 314.54 = ATCC 11832, from kerion of human scalp, C.A. Fuentes, June 1954. Zoophilic species on pigs; human inflammatory infections occur.

Fac. syn.: *Nannizzia obtusa* Dawson & Gentles, Sabouraudia 1: 56, 1961 ≡ *Arthroderma obtusum* (Dawson & Gentles) Weitzman, McGinnis, Padhye & Ajello, Mycotaxon 25: 514, 1986. Type: crossing of strains IMI 117073 (MT+) × IMI 117064 (MT−) (mating strains CBS 321.61, CBS 322.61), from human patient, C.O. Dawson and J.C. Gentles.

**8.** ***Nannizzia persicolor*** (Sabouraud) Stockdale; *Trichophyton persicolor* Sabouraud, Malad. Cuir Chev. 3: 632, 1910 ≡ *Ectotrichophyton persicolor* (Sabouraud) Castellani & Chalmers, Man. Trop. Med. p. 1005, 1918 ≡ *Sabouraudites persicolor* (Sabouraud) Ota & Langeron, Annls Parasit. Hum. Comp. 1: 329, 1923 ≡ *Closteroaleuriosporia persicolor* (Sabouraud) Grigorakis, Annls Sci. Nat., Bot., Sér. 10, 7: 412, 1925 ≡ *Microsporum persicolor* (Sabouraud) Guiart & Grigorakis, Lyon Méd. 141: 377, 1928 ≡ *Ctenomyces persicolor* (Sabouraud) Nannizzi, Tratt. Micopat. Um. 4: 154, 1934 ≡ *Epidermophyton persicolor* (Sabouraud) C.W. Dodge, Med. Mycol. p. 486, 1935 ≡ *Langeronites persicolor* (Sabouraud) Ansel 1957 ≡ *Trichophyton mentagrophytes* (Robin) Blanchard var. *persicolor* (Sabouraud) Ueckert, Zentbl. Bakt. Parasitkde, Abt. 1, 176: 127, 1959 ≡ *Nannizzia persicolor* (Sabouraud) Stockdale, Sabouraudia 5: 357, 1967 ≡ *Microides persicolor* (Sabouraud) De Vroey, Ann. Soc. Belg. Méd. Trop. 50: 25, 1970 ≡ *Arthroderma persicolor* (Sabouraud) Weitzman, McGinnis, Padhye & Ajello, Mycotaxon 25: 514, 1986. The original material of Sabouraud is not known to be preserved. The name is defined by the teleomorph described by Stockdale [65] which is here taken to be meant as a new combination. The respective dried material is therefore a **neotype designated herewith:** IMI 126886, cross of living strains IMI 117073 (MT+), from bank vole, UK × IMI 117064 (MT−), from shrew, UK, M.E. English. Zoophilic species.

Fac. syn.: *Nannizzia quinckeani* Balabanov & Schick, Derm. Venereol. 9: 35, 1970. Type strain: CBS 871.70, from human skin, Bulgaria, V.A. Balabanov.

**9.** ***Nannizzia praecox*** (Padhye, Ajello & McGinnis) Gräser & de Hoog, **comb. nov.**

Basionym: *Sabouraudites praecox* Rivalier, Annls Inst. Pasteur 86: 276, 1954 (invalid) ≡ *Microsporum praecox* (Rivalier) Rivalier, Bull. Soc. Fr. Mycol. Méd. 7: 297, 1978 (invalid) ≡ *Microsporum praecox* Rivalier ex Padhye, Ajello & McGinnis, *in* Padhye, Detweiler, Frumkin, Bulmer, Ajello & McGinnis, J. Med. Vet. Mycol. 27: 316, 1989. Holotype CDC B-4819D; authentic strain CBS 288.55, from human, E. Rivalier.

### Clade D: *Paraphyton* Gräser, Dukik & de Hoog, gen. nov

Colonies mostly granular, brownish, with a brown colony reverse. Hyphae thin-walled, hyaline. Thallic macroconidia and microconidia, if present, arranged in orthotropically arranged hyphal systems. Macroconidia, multi-celled, thick- and rough-walled, (sub)hyaline, clavate or cigar-shaped. Microconidia thin- and smooth-walled, hyaline, 1-celled, clavate. Sexual state produced after mating, arthroderma-like.

Type species: *Microsporum cookei* Ajello.

**1.** ***Paraphyton cookei*** (Ajello) Gräser, Dukik & de Hoog, **comb. nov.**

Basionym: *Microsporum cookei* Ajello, Mycologia 51: 71, 1959. Type strain: CBS 228.58 = CDC B-276, from soil, Kentucky, USA, L. Ajello. Geophilic species.

Fac. syn.: *Nannizzia cajetani* Ajello, Sabouraudia 1: 175, 1961 ≡ *Arthroderma cajetani* (Ajello) Ajello, Weitzman, McGinnis & Padhye, *in* Weitzman, McGinnis, Padhye & Ajello, Mycotaxon 25: 513, 1986. Neotype: CDC B-4218; type strain: ATCC 14386 = CDC B-433 [crossing of ATCC 14387 (MT+) × ATCC 14388 (MT−)], from soil, Michigan, USA, L. Ajello.

Fac. syn.: *Microsporum racemosum* Borelli, Acta Méd. Venez. 12: 150, 1965 ≡ *Nannizzia racemosa* (Borelli) Rush-Munro, J.M.B. Smith & Borelli, Mycologia 62: 858, 1970 ≡ *Arthroderma racemosa* (Rush-Munro, J.M.B. Smith & Borelli) Weitzman, McGinnis, Padhye & Ajello, Mycotaxon 25: 514, 1986. Holotype: IMI 128984, crossing of CBS 424.74 = ATCC 18911 = CDC X-903 = IHEM 3452 = IMI 135823 = NCPF 484 = UAMH 3368 (MT−) × CBS 423.74 = ATCC 18910 = CDC X-902 = IHEM 3453 = IMI 135822 = NCPF 483 = UAMH 3367 (MT+), from soil, Georgia, USA, A.A. Padhye.

**2.** ***Paraphyton cookiellum*** (de Clerq) Gräser, Dukik & de Hoog, **comb. nov.**

Basionym: *Nannizzia cookiella* de Clercq, Mycotaxon 18: 24, 1983 ≡ *Arthroderma cookiellum* (de Clercq) Weitzman, McGinnis, Padhye & Ajello, Mycotaxon 25: 513, 1986. Holotype CBS-H 7397, type strains: CBS 101.83 (MT−) × CBS 102.83 (MT+), from soil, Abidjan, Ivory Coast, D. de Clercq, 1984. Geophilic species.

**3.** ***Paraphyton mirabile*** (J.S. Choi, Gräser, Walther, Peano, Symoens & de Hoog) Gräser, Dukik & de Hoog, **comb. nov.**

Basionym: *Arthroderma mirabile* J.S. Choi, Gräser, Walther, Peano, Symoens & de Hoog, Med. Mycol. 50: 168, 2012 ≡ *Microsporum mirabile* J.S. Choi, Gräser, Walther, Peano, Symoens & de Hoog, Med. Mycol. 50: 168, 2012. Holotype CBS H-20571, cross of CBS 124422 = IHEM 24407, from pelt of wild chamois, Italy, A. Peano (MT+) × CBS 129179 = IHEM 24409, from human toenail, The Netherlands (MT−). Zoophilic species [66].

### Clade E: *Lophophyton*

Colonies expanding, granular or velvety, with brownish to red pigments. Macroconidial in loose clusters, large, up to 60 μm in length, thick- and rough-walled, multiseptate. Microconidia present. Sexual state produced after mating, arthroderma-like.

Type species: *Epidermophyton gallinae* Mégnin.

**1.** ***Lophophyton gallinae*** (Mégnin) Matruchot & Dassonville; *Epidermophyton gallinae* Mégnin, C. R. Soc. Biol. 33: 404, 1881 ≡ *Lophophyton gallinae* (Mégnin) Matruchot & Dassonville, Revue Gén. Bot. 11: 429, 1899 ≡ *Achorion gallinae* (Mégnin) Sabouraud, Malad. Cuir Chev. 3: 553, 1910 ≡ *Sabouraudites gallinae* (Mégnin) Ota & Langeron, Annls Parasit. Hum. Comp. 1: 327, 1923 ≡ *Closteroaleuriospora gallinae* (Mégnin) Grigorakis, Annls. Sci. Nat., Bot., Sér. 10, 7: 412, 1925 ≡ *Microsporum gallinae* (Mégnin) Grigoraki, Annls Derm. Syph., Sér. 6, 10: 42, 1929 ≡ *Trichophyton gallinae* (Mégnin) Georg, Mycologia 44: 486, 1952. **Neotype designated herewith:** CBS 300.52, F. Blank. Zoophilic species on poultry.

Fac. syn.: *Nannizzia grubyi* Georg, Ajello, Friedman & Brinkman, Sabouraudia 1: 194, 1962 ≡ *Arthroderma grubyi* (Georg, Ajello, Friedman & Brinkman) Ajello, Weitzman, McGinnis & Padhye, *in* Weitzman, McGinnis, Padhye & Ajello, Mycotaxon 25: 513, 1986. Neotype: CDC B-4219 (= CDC X-322 = CBS 243.66 = ATCC 14419 = IMI 113720 = NCPF 487, from dog ringworm, Missouri, USA, A.E. Blum × CDC X-321, from human ringworm, USA, L. Friedman. Reference strains: ATCC 14422 = CDC X-470 = CBS 100083 (MT+) × ATCC 14423 (MT−), single ascospore isolates from cross of CBS 243.66 × CDC X-321.

Fac. syn.: *Microsporum vanbreuseghemii* Georg, Ajello, Friedman & S.A. Brinkman, Sabouraudia 1: 191, 1961/62. Type strain: CBS 243.66 = ATCC 14419 = CDC X-322 = IMI 113720 = NCPF 487, from dog ringworm, Missouri, USA, A.E. Blum.

### Clade F: *Microsporum*

Colonies mostly granular to cottony, yellowish to brownish, with a cream-colored or brown colony reverse. Hyphae thin-walled, hyaline. Thallic macroconidia and microconidia, if present, arranged in orthotropically arranged hyphal systems. Macroconidia, multi-celled, thick- and rough-walled, (sub)hyaline, clavate, fusiform or cigar-shaped. Microconidia thin- and smooth-walled, hyaline, 1-celled, clavate. Sexual state sometimes produced after mating, arthroderma-like. Note that the three currently accepted species cannot be reliably distinguished by multilocus sequence analysis (Fig. 5); more detailed species are needed to establish species borderlines.

Type species: *Microsporum audouinii* Gruby.

**1.** ***Microsporum audouinii*** Gruby, C. R. Hebd. Séanc. Acad. Sci., Paris 17: 301, 1843 ≡ *Sporotrichum audouinii* (Gruby) Saccardo, Syll. Fung. 4: 101, 1886 ≡ *Sabouraudites audouinii* (Gruby) Ota & Langeron, Annls Parasit. Hum. Comp. 1: 327, 1923 ≡ *Closteroaleurosporia audouinii* (Gruby) Grigoraki, Annls Sci. Nat., Bot., Sér. 10, 7: 412, 1925 ≡ *Veronaia audouinii* (Gruby) Benedek, Mycopath. Mycol. Appl. 14: 115, 1961. Neotype strain: CBS 545.93, from human skin, The Netherlands. Anthropophilic species.

Fac. syn.: *Sabouraudites langeronii* Vanbreuseghem, Annls Parasit. Hum. Comp. 25: 516, 1950. Authentic strain: CBS 404.61, from human, Zaire, R. Vanbreuseghem.

**2.** ***Microsporum canis*** (Bodin) Bodin; *Microsporum audouinii* Gruby var. *canis* Bodin, *in* Besnier, Brocq & Jacquet, Prat. Derm. p. 810, 1900 ≡ *Microsporum canis* (Bodin) Bodin, Champ. Paras. Homme p. 137, 1902 ≡ *Sabouraudites canis* (Bodin) Langeron, Précis Mycol. p. 534, 1945. Neotype strain: CBS 496.86, from feline ringworm, Japan, M. Hironaga. Zoophilic species.

Fac. syn.: *Nannizzia otae* Hasegawa & Usui, Jpn. J. Med. Mycol. 16: 151, 1975 ≡ *Arthroderma otae* (Hasegawa & Usui) McGinnis, Weitzman, Padhye & Ajello, *in* Weitzman, McGinnis, Padhye & Ajello, Mycotaxon 25: 514, 1986. Holotype: VMUT-1, cross of monascospore cultures VUT-73015 = ATCC 28327 × VUT-74001 = ATCC 28328; reference strains CBS 495.86 = VUT-77054 (MT+) × CBS 496.86 = VUT 77055 (MT−), from feline ringworm, Japan, M. Hironaga. Note that the two mating partners are rather remote from each other, syntypes being located in *M. canis* and *M. audouinii* clusters (Fig. 5).

Fac. syn.: *Microsporum distortum* di Menna & Marples, Trans. Br. Mycol. Soc. 37: 372, 1954 ≡ *Microsporum canis* Bodin var. *distortum* (di Menna & Marples) Matsumoto, Padhye & Ajello, Trans. Br. Mycol. Soc. 81: 649, 1983. Type strain: CBS 101514 = NCPF 215, from human tinea capitis, New Zealand.

**3.** ***Microsporum ferrugineum*** Ota, Jpn. J. Derm. Urol. 21: 201, 1921 ≡ *Grubyella ferruginea* (Ota) Ota & Langeron, Annls Parasit. Hum. Comp. 1: 330, 1923 ≡ *Arthrosporia ferruginea* (Ota) Grigoraki, Annls Sci. Nat., Bot., Sér. 10, 7: 414, 1925 ≡ *Achorion ferrugineum* (Ota) Guiart & Grigoraki, Lyon Méd. 141: 377, 1928 ≡ *Trichophyton ferrugineum* (Ota) Talice, Annls Parasit. Hum. Comp. 9: 83, 1931. Authentic strain: CBS 497.48, from human skin, Japan, M. Ota. Anthropophilic species.

### Clade G: *Arthroderma*

Colonies mostly granular to cottony, yellowish to brownish, with a cream-colored or brown colony reverse. Hyphae thin-walled, hyaline. Thallic macroconidia and microconidia, if present, arranged in orthotropically arranged hyphal systems. Macroconidia, multi-celled, thick- and rough-walled, (sub)hyaline, clavate, fusiform or cigar-shaped. Microconidia thin- and smooth-walled, hyaline, 1-celled, clavate. Sexual state sometimes produced after mating, arthroderma-like.

Type species: *Arthroderma curreyi* Berkeley.

**1.** ***Arthroderma amazonicum*** (Moraes, Borelli & Feo) Gräser & de Hoog, **comb. nov.**

Basionym: *Microsporum amazonicum* Moraes, Borelli & Feo, Med. Cután. 11: 284, 1967. Type strain: CBS 967.68 = ATCC 18393, from hair of *Oryzomys* rat, Manaus, Brazil.

Fac. syn.: *Nannizzia borellii* Moraes, Padhye & Ajello, Mycologia 67: 1112, 1976 ≡ *Arthroderma borellii* (Moraes, Padhye & Ajello) Padhye, Weitzman, McGinnis & Ajello, *in* Weitzman, Mycotaxon 25: 513, 1986. Holotype CDC B-2093, cross of CDC B-2087 × B-2089, reference strains CBS 221.75 = ATCC 28356 = CDC Y-81 = IHEM 3454 (MT+) × ATCC 28357 = CDC Y-82 = IHEM 3455 (MT−), all from fur of spiny rat (*Proechimys guannensis*), Belém, Brazil. Zoophilic species. The species is located in an ancestral position to the *Arthrodermataceae*; its taxonomy requires further study.

**2.** ***Arthroderma ciferrii*** Varsavsky & Ajello; *Trichophyton georgiae* Varsavsky & Ajello, Riv. Patol. Veg., Pavia, Sér. 3, 4: 357, 1964 ≡ *Arthroderma ciferrii* Varsavsky & Ajello, Riv. Patol. Veg., Pavia, Sér. 3, 4: 358, 1964 ≡ *Chrysosporium georgiae* (Varsavsky & Ajello) v. Oorschot, Stud. Mycol. 20: 31, 1980. Type strain: CBS 272.66 = UAMH 2534, from soil, Arkansas, USA, L. Ajello. Geophilic species.

**3.** ***Arthroderma cuniculi*** Dawson, Sabouraudia 2: 187, 1963. Holotype: IMI 96243, cross of single ascospore strains CBS 492.71 = ATCC 28442 = IHEM 4437 = IMI 96244 = NCPF 525A (MT−), from soil and hair of rabbit, Scotland, UK, C.O. Dawson × CBS 495.71 = ATCC 18444 = IMI 96245 (MT+), from soil and hair of rabbit, Scotland, UK, C.O. Dawson. Geophilic species.

**4.** ***Arthroderma curreyi*** Berkeley, Outl. Brit. Fung. p. 357, 1860. **Epitype, designated herewith:** CBS 353.66, from dune soil, UK, A.E. Apinis, 1966. Geophilic species.

**5.** ***Arthroderma eboreum*** (Brasch & Gräser) Gräser & de Hoog, **comb. nov.**

Basionym: *Trichophyton eboreum* Brasch & Gräser, J. Clin. Microbiol. 43: 5235, 2005. Type strain: CBS 117155 = DSM 16978, from human skin, Ivory Coast, J. Brasch.

Fac. syn.: *Arthroderma olidum* Cambell, Borman, Linton, Bridge & Johnson, Med. Mycol. 44: 457, 2006. Holotype: NCPF 5111, type strain NCPF 5088, crossing of NCPF 5102 × NCPF 5104, from badger hole soil, UK

**6.** ***Arthroderma flavescens*** R.G. Rees, Sabouraudia 5: 206, 1967 ≡ *Trichophyton flavescens* Padhye & Carmichael, Can. J. Bot. 49: 1535, 1971. Type strain: IMI 117342, crossing of IMI 112079, from feather of lorikeet (*Trichoglossus moluccanus*), Queensland, Australia, R.G. Rees, × IMI 117341 = CBS 473.78, from feather of sacred kingfisher (*Halycon sancta*), Queensland, Australia, R.G. Rees. The anamorph was later introduced for one of the strains producing the teleomorph. Zoophilic species.

**7.** ***Arthroderma gertleri*** Böhme, Mykosen 10: 251, 1967. Type: UAMH 2620, from soil, Germany, H. Böhme. Geophilic species.

Fac. syn.: *Trichophyton vanbreuseghemii* Rioux, Jarry & Juminez, Nat. Monspeliensia, Sér. Bot. 16: 158, 1964 (*non Arthroderma vanbreuseghemii* Takashio, Ann. Soc. Belg. Méd. Trop. 53: 547, 1973). Type strain: CBS 598.66, from soil, J.A. Rioux. The oldest name for this taxon is *T. vanbreuseghemii*, but the combination cannot be made because of an earlier homonym.

**8.** ***Arthroderma gloriae*** Ajello & Cheng; *Trichophyton gloriae* Ajello, *in* Ajello & Cheng, Mycologia 59: 257, 1967 ≡ *Arthroderma gloriae* Ajello & Cheng, Mycologia 59: 257, 1967. Type strain anamorph: CBS 228.79 = CDC X-138 = ATCC 16655, type strain teleomorph: crossing of CBS 664.77 = CDC X779 = UAMH 2820 = ATCC 16657 (MT+) × CBS 663.77 = CDC X780 = ATCC 16658 (MT−), from soil, Arizona, USA Geophilic species.

**9.** ***Arthroderma insingulare*** Padhye & Carmichael, Sabouraudia 10: 49, 1972. Reference strains: CBS 521.71 = ATCC 22519 = UAMH 3441 (MT A), from soil, Alberta, Canada, A.A. Padhye; CBS 522.71 = ATCC 22520 = IMI 158874 = NCPF 470 = UAMH 3442 (MT a), from soil, Alberta, Canada, A.A. Padhye.

**10.** ***Arthroderma lenticulare*** Pore, Tsao & Plunkett, Mycologia 57: 970, 1965. Reference strains: CBS 307.65 = ATCC 18445 = IHEM 3717 (MT+) × CBS 308.65 = ATCC 18446 = IHEM 3703 (MT−), both from soil of gopher hole, Los Angeles County, USA, R.S. Pore. Geophilic species.

**11.** ***Arthroderma melis*** Křivanec, Janečková & Otčenášek, Česká Mykol. 31: 92, 1977. Type strain: CBS 669.80, from burrow of badger (*Melis melis*), Moravia, Czech Republic. Geophilic species; no growth at 37 °C.

**12.** ***Arthroderma multifidum*** Dawson, Sabouraudia 2: 189, 1963. Syntype strains: CBS 419.71 = ATCC 18440 = IHEM 4432 = IMI 094205 (MT+) × CBS 420.71 = ATCC 18441 = IMI 094206 (MT−), both from soil and hair from rabbit burrow, UK, C.O. Dawson. Geophilic species.

**13.** ***Arthroderma onychocola*** **(**Cmokova, Hubka, Skorepova & Kolařík) Gräser & de Hoog, **comb. nov.**

Basionym: *Trichophyton onychocola* Cmokova, Hubka, Skorepova & Kolařík, Med. Mycol. 52: 287, 2014. Type strain: CBS 132920, from human nail, Czechia. Anthropophilic species [67].

**14.** ***Arthroderma phaseoliforme*** (Borelli & Feo) Gräser & de Hoog, **comb. nov.**

Basionym: *Trichophyton phaseoliforme* Borelli & Feo, Acta Méd. Venez. 13: 176, 1966. Type strain: CBS 364.66, from pelt of mountain rat (*Proechimys guyanensis*), Venezuela. Geophilic species [68].

**15.** ***Arthroderma quadrifidum*** Dawson & Gentles, Sabouraudia 1: 35, 1961. Type not indicated; authentic strains CBS 117.61 (MT+) × CBS 118.61 (MT−), sent by C.O. Dawson & J.C. Gentles, 1961. Geophilic species.

**16.** ***Arthroderma redellii*** (Minnis, Lorch, D.L. Lindner & Blehert) Gräser & de Hoog, **comb. nov.**

Basionym: *Trichophyton redellii* Minnis, Lorch, D.L. Lindner & Blehert, *in* Lorch, Minnis, Meteyer, Redelli, White, Kaarakka, Muller, Lindner, Verant, Shearn-Bochsler & Blehert, J. Wildlife Dis. 51: 43, 2015. Type strain: CBS 134551 = CFMR 44738-03H, wing of hibernating bat (*Myotis lucifugus*), Wisconsin, USA, M.L. Verant, February 2012. Zoophilic species.

**17.** ***Arthroderma silverae*** Currah, S.P. Abbott & Sigler, Mycol. Res. 100: 195, 1996. Type: UAHM 6517 [69]. The strain was not available for study.

**18.** ***Arthroderma thuringiensis*** (Koch) Gräser & de Hoog, **comb. nov.**

Basionym: *Trichophyton thuringiense* Koch, Mykosen 12: 288, 1969. Type strain: CBS 417.71 = ATCC 22648 = IMI 134993 = NCPF 492A = UAMH, from mouse skin, Germany, H.A. Koch, 1964. Zoo- or geophilic species [7].

**19.** ***Arthroderma tuberculatum*** Kuehn, Mycopath. Mycol. Appl. 13: 190, 1960. Type strain: CBS 473.77 = ATCC 26700 = UAMH 873, feather of *Turdus americanus*, Illinois, USA, H.H. Kuhn. Geophilic species.

**20.** ***Arthroderma uncinatum*** Dawson & Gentles, Sabouraudia 1: 55, 1961. Syntypes: CBS 315.65 (MT+), CBS 316.65 (MT−), both from soil, California, USA, O.A. Plunkett. Geophilic species.

Fac. syn.: *Keratinomyces ajelloi* Vanbreuseghem, Bull. Acad. R. Méd. Belg. 38: 1075, 1952 ≡ *Epidermophyton terrigenum* Evolceanu & Alteras, Mycopath. Mycol. Appl. 11: 202, 1959 (name change) ≡ *Microsporum ajelloi* (Vanbreuseghem) Arievitch & Stiepanishchewa, Proc. Int. Symp. Med. Mycol., Warsaw p. 43, 1965 ≡ *Trichophyton ajelloi* (Vanbreuseghem) Ajello, Sabouraudia 6: 148, 1966 ≡ *Epidermophyton ajelloi* (Vanbreuseghem) Novák & Galgóczy, Acta Bot. Hung. 15: 130, 1969. Type strain: CBS 101515 = NCPF 216, from soil, Belgium, R. Vanbreuseghem.

Fac. syn.: *Epidermophyton stockdaleae* Prochacki & Engelhardt-Zasada, Mycopathologia 54: 342, 1974. Type strain: CBS 128.75, from soil, Poland, C. Engelhardt.

Fac. syn.: *Keratinomyces ajelloi* Vanbreuseghem var. *nanum* Kunert & Hejtmánek, Česká Epid. Mikrobiol. Immunol. 13: 296, 1964 ≡ *Trichophyton ajelloi* (Vanbreuseghem) Ajello var. *nanum* (Kunert & Hejtmánek) Ajello, Sabouraudia 6: 148, 1966. Type strain: CBS 180.64 = ATCC 22398 = NCPF 473, from soil, Czechoslovakia, M. Hejtmánek, 1964.

**21.** ***Arthroderma vespertilii*** **(**Guarro, Vidal & De Vroey) Gräser & de Hoog, **comb. nov.**

Basionym: *Chrysosporium vespertilii* Guarro, Vidal & De Vroey, *in* Vidal, Guarro & De Vroey, Mycotaxon 59: 190, 1996. Type strain: CBS 355.93 = IMI 357403 = FMR 3752, from intestinal content of bat, Kibisi, near Kinshasa, Zaire [70]. Zoophilic species.

### List of Doubtful Dermatophyte Names Not Listed as Such in *Atlas of Clinical Fungi*

***castellanii***—*Veronaia castellanii* Benedek, Mycopath. Mycol. Appl. 14: 115, 1961. Type material not known to be preserved; identity doubtful.

***ceretanicus***—*Keratinomyces ceretanicus* Punsola & Guarro, Mycopathologia 85: 185, 1984.

Type strain: CBS 269.89 = FMR 3063, from soil, Valdivia, Chile, J. Guarro, Nov. 1988. The type species of *Keratinomyces, K. ajelloi* clusters in *Arthroderma*. *Keratinomyces ceretanicus* is a phylogenetically distant, psychrophilic soil fungus in the *Onygenace*. We propose the following, as yet monotypic genus for this fungus:

***Guarromyces*** Gräser & de Hoog, **gen. nov.**

Macroconidia hyaline, smooth- and thick-walled, lanceolate to cylindrical, multiseptate, borne holothallically in loose clusters on creeping hyphae; microconidia absent. Type species: ***Guarromyces ceretanicus*** (Punsola & Guarro) Gräser & de Hoog, **comb. nov.**

***granulosum***—*Trichophyton granulosum* Sabouraud, *in* Pécus, Rev. Gén. Méd. Vét. 15: 561, 1909 ≡ *Trichophyton mentagrophytes* (Robin) Blanchard var. *granulosum* (Sabouraud) Neveu-Lemaire, Précis Parasitol. Anim. Domest. p. 71, 1912 ≡ *Ectotrichophyton granulosum* (Sabouraud) Castellani & Chalmers, Man. Trop. Med., ed. 3, p. 1006, 1919 ≡ *Sabouraudites granulosus* (Sabouraud) Ota & Langeron, Annls Parasit. Hum. Comp. 1: 328, 1923 ≡ *Chlamydoaleuriospora granulosa* (Sabouraud) Grigoraki, Annls Sci. Nat., Bot., Sér. 10, 7: 412, 1925 ≡ *Trichophyton gypseum* Bodin var. *granulosum* (Sabouraud) Frágner, Česká Mykol. 10: 108, 1956. In general this species is treated as a heavily sporulating variant of *T. mentagrophytes* occurring on cats and dogs [52]. As no type material is known to be preserved, its identity remains doubtful.

***gypseum***—*Trichophyton gypseum* Bodin, Champ. Paras. Homme p. 115, 1902 ≡ *Achorion gypseum* (Bodin) Bodin, Annls Derm. Syph. 4: 585, 1907 ≡ *Sabouraudites gypseus* (Bodin) Ota & Langeron, Annls Parasit. Hum. Comp. 1: 328, 1923 ≡ *Closterosporia gypsea* (Bodin) Grigoraki, Annls Sci. Nat., Bot., Sér. 10, 7: 411, 1925 ≡ *Microsporum gypseum* (Bodin) Guiart & Grigoraki, Lyon Méd. 141: 377, 1928 ≡ *Trichophyton mentagrophytes* (Robin) Blanchard var. *gypseum* (Bodin) Kamyszek, Med. Weteryn. 24: 146, 1945. Type material not known to be preserved; doubtful species.

***lanosa***—*Closterosporia lanosa* Grigoraki, C. R. Hebd. Séanc. Acad. Sci., Paris 179: 1424, 1924. Type material not known to be preserved; doubtful species.

***microsporum***—*Oidium microsporium* Kambayashi, Jpn. J. Derm. Urol. 21: 460, 1921. Type material not known to be preserved; identity doubtful.

***serratus***—*Ctenomyces serratus* Eidam, Eitr. Biol. Pfl. 3: 274, 1880. *Ctenomyces* is a gymnothecial genus of terrestrial fungi with chrysosporium-like conidia and is classified in the *Gymnoascaceae*. Several species have been classified in the genus. For a description, see Böhme [71].

***terrestre***—*Trichophyton terrestre* Durie & Frey, Mycologia 49: 401, 1957. Type UAMH was not available for study. In literature the species has been listed as the anamorph of different *Arthroderma* species which on molecular grounds appear to be remote from each other. *Trichophyton terrestre* needs to be reevaluated.

***terrestre*** **-** ***primum***—*Trichophyton terrestre*-*primum* Szathmáry, Magya Orvosi Arch. 37-6: 1–6, 1936. Type material not known to be preserved; identity doubtful.

### Epilogue

The present paper provides an evaluated list of currently accepted species in *Arthrodermataceae*, but is by no means exhaustive. Many groups require more detailed polyphasic studies with mating experiments to determine exact borderlines between species. Some extant types could not be acquired during the course of this study. New, genomic and proteomic studies will provide understanding of the observed clinical differences in predilection between closely related species. It is expected that among the geo- and zoophilic groups numerous species are yet to be discovered in undersampled habitats; our review means to provide a new starting point for these subsequent studies.

## Electronic supplementary material

Below is the link to the electronic supplementary material.

Supplementary material 1 (XLSX 23 kb)
